# Application of the HPLC-ELSD technique for the determination of major metabolites of ibuprofen and creatinine in human urine

**DOI:** 10.1038/s41598-023-47594-8

**Published:** 2023-11-20

**Authors:** Justyna Piechocka, Natalia Matwiej, Marta Gaweł, Michał Matyjaszczyk, Rafał Głowacki, Grażyna Chwatko

**Affiliations:** 1 Faculty of Chemistry, Department of Environmental Chemistry, University of Lodz, Pomorska 163/165, 90-236 Lodz, Poland; 2 Doctoral School of Exact and Natural Sciences, University of Lodz, Banacha 12/16, 90-237 Lodz, Poland; 3 Department of Family Medicine, Polish Mother’s Memorial Hospital Research Institute, Rzgowska 281/289, 93-338 Lodz, Poland; 4 Department of Family Medicine, Medical University of Lodz, Narutowicza 60, 90-131 Lodz, Poland

**Keywords:** Bioanalytical chemistry, Medical and clinical diagnostics

## Abstract

The report presents robust and high throughput methods, based on liquid chromatography coupled with evaporative light scattering detection (HPLC-ELSD), for the simultaneous determination of major metabolites of ibuprofen (IBU), namely 2-hydroxyibuprofen and carboxyibuprofen (method A) as well as creatinine (Crn) (method B) in human urine. The assays primarily involve straightforward sample purification. For both methods, the chromatographic separation of the analytes is achieved within 8 min at room temperature on Poroshell 120 SB-C18 (75 × 4.6 mm, 2.7 µm) column using gradient elution. The eluents consisted of 0.1% formic acid in water and acetonitrile (method A) or water and methanol (method B) delivered at a flow rate of 1 or 0.5 mL/min, respectively. In relation to metabolites of IBU, the assay linearity was observed within 0.06–0.5 g/L in urine, while the Crn assay linearity was demonstrated within 0.5–30 mmol/L in urine. The limit of quantification for IBU metabolites was determined to be 0.06 g/L, and 0.5 mmol/L for Crn. These methods were successfully applied to urine samples delivered by ten apparently healthy donors showing that the HPLC-ELSD assays are suitable for human urine screening.

## Introduction

Ibuprofen (IBU) is one of the most commonly used non-steroidal anti-inflammatory, analgesic, and antipyretic drug in human and veterinary medicine. In humans, the drug is rapidly absorbed after ingestion and is extensively metabolized to inactive compounds, namely 1-hydroxyibuprofen, 2-hydroxyibuprofen (2-HIBU), 3-hydroxyibuprofen, carboxyibuprofen (CIBU) and occurring in a trace amounts their glucuronic acid conjugates^1,2^. Pharmacokinetic studies have shown that the majority of the given dose of IBU is metabolized and eliminated within 24 h in urine. Only a trace amount of the drug is excreted in urine in the unchanged form^1,2^. Unfortunately, IBU is sometimes misused, most probably as it is an over-the-counter drug. Since adverse effects of IBU are widely known^1–4^, facile methods for the determination of the IBU in the body and more importantly its metabolites in urine are necessary. These tools could help in the case of diagnostic exclusion of acute overdose or chronic drug abuse. In parallel, it is also essential to control creatinine (Crn) level in study samples, a compound that is inherently present in all urine samples and widely accepted as a reference for sample normalization in diagnostic testing^5,6^.

Numerous methods for determining the above-mentioned compounds have been developed so far, and many review articles on Crn^7–9^ and IBU^10–13^ are available. In general, these assays are predominantly based on separation techniques which are used for the determination of such compounds in complex systems. These methods have high-throughput potential, sensitivity, specificity, and excellent resolution, along with high degrees of reproducibility and accuracy. Interestingly, many assays for the quantification of IBU have been elaborated on^10–13^, whereas only a few methods allow simultaneous determination of IBU and its metabolites in urine^14–25^. Among them, methods based on liquid chromatography (HPLC)^16,17,19,21,23,24^, gas chromatography (GC)^14,15,18^ and capillary electrophoresis (CE)^20,22,25^ coupled with ultraviolet (UV)^16,17,20,22,23,25^, fluorescence^24^, nuclear magnetic resonance^21^ and mass spectrometry (MS)^14,15,18,19^ detection have been proved to be suitable for fulfilling the purpose. Interestingly, none of these methods allow for the determination of IBU and its metabolites as well as Crn using HPLC coupled with evaporative light scattering detection (ELSD). ELSD is a low-cost and general-purpose universal detector that can identify semi- and non-volatile analytes over a wide dynamic range with uniform sensitivity regardless of their spectroscopic properties^26–29^. In addition, the existing approaches^14–25^ are challenging because they involve relatively labor-intensive, time/energy-consuming, and sophisticated sample processing procedures. In fact, the sample workup is a multistep process consisting primarily of enzymatic or alkaline hydrolysis^14,17,19–25^, extensive sample purification by multiple extraction procedures, such as liquid–liquid (LLE)^14,15,17,18,24^, solid phase (SPE)^15,16,20–23,25^, ultrasound-assisted emulsification (USAEME)^19^ (micro)extraction and chemical derivatization^14,15,18,24^. The use of these multiple steps can result in an increase in the amount of consumed plastic disposable materials and chemicals as well as considerable risk of losing analyte and precision diminution, among others. Overall, there is undoubtedly a need for streamlining the procedures and providing more effective analytical tools.

As a result, the present paper aims to provide evidence that the practical application of HPLC-ELSD technique can be extended to IBU, 2-HIBU, CIBU, and Crn identification. The method enables the simultaneous determination of IBU and its main metabolites, namely 2-HIBU and CIBU (method A) as well as Crn (method B) in human urine. An additional objective of research work was related to greening analytical methodologies and making everything as simple as possible. Urine was selected as the matrix of choice, as it is easily accessible and can be obtained in a non-intrusive and non-invasive way; and because it has been demonstrated that in humans, the IBU and products of its biotransformation are mainly excreted in urine^1,2^. Important milestones included the development of an effective analytical tools based on HPLC-ELSD and application to real samples in order to demonstrate the methods performance.

## Materials and methods

### Reagents and materials

All chemicals were commercially available and at least of analytical-reagent grade. Crn, IBU sodium salt, 2-HIBU, CIBU as well as HPLC-gradient grade acetonitrile (ACN) and methanol (MeOH) were obtained from Sigma-Aldrich (St. Louis, MO, USA). Mobile phase additive suitable for HPLC–MS technique, namely formic acid (FA) was purchased from Merck KGaA (Darmstadt, Germany), while perchloric acid (PCA) was from J.T. Baker (Deventer, The Netherlands). Deionized water was produced in the laboratory. Commercially available 400 mg or 600 mg tablets containing active substance IBU, were used.

### Instrumentation

All analyses were performed using an Agilent 1220 Infinity LC system equipped with a binary pump integrated with a two-channel degasser, autosampler, column oven, UV detector, and ELSD detector 1260 Infinity II series from Agilent Technologies (Waldbronn, Germany). Instrument control, data acquisition, and analysis were carried out using OpenLAB CDS software. Analytes were separated on Poroshell 120 SB-C18 (75 × 4.6 mm, 2.7 µm) column from Agilent Technologies (Waldbronn, Germany). During the study, a Mikro 220R centrifuge with a fast cool function (Hettich Zentrifugen, Tuttlingen, Germany), and Multi-Speed Vortex MSV-3500 (Biosan, Riga, Latvia) were used. Samples were stored in an ultra-low-temperature freezer (Panasonic Healthcare Co., Ltd., Sakata, Japan). Water was purified using a Direct-Q 3 UV water purification system (Millipore, Vienna, Austria).

### Stock solutions

The stock solutions of IBU (10 g/L), 2-HIBU (1 g/L), and CIBU (1 g/L) were prepared by dissolving an appropriate amount of the powder in a mixture of MeOH and water (50:50, *v/v*). These solutions were kept at − 20 °C for no longer than 7 days without a noticeable change in the analyte content. The working solutions were prepared daily by diluting a particular standard solution with a mixture of MeOH in water (50:50, *v/v*) as needed and processed without delay.

The stock solution of Crn (0.15 mol/L) was prepared as needed in deionized water. The solution was kept at 4 °C for a maximum of 7 days without a noticeable change in the analyte content. The working solutions of Crn were prepared daily by diluting a particular standard solution with deionized water as needed and processed without delay.

The remaining solutions, including PCA (3 mol/L) and mobile phase component consisting of 0.1% FA in water were prepared by diluting a particular standard solution with deionized water and stored in tight glass flasks at ambient temperature.

### Biological samples collection

First, early morning urine samples (about 10 mL) were collected from individuals after overnight fasting using a standard method^5^. Samples of “mid-stream” urine were obtained by asking donors to put fluid into a sterile container. Then, samples were cooled on ice, delivered to the laboratory within 3 h after collection, and stored at − 80 °C until analysis. In each case, samples were processed without delay, immediately after defrosting at room temperature, using the procedures described in "Urine samples preparation" and "Chromatographic conditions" sections.

A group of ten apparently healthy anonymous individuals was involved in the study. The control subjects, belonging to an ethnically homogeneous group, were neither supplemented with the analytes before sample collection, or their precursors for at least 7 days prior to sampling. In addition, no medications were allowed. Regarding donors who have administered one dose of IBU (400 mg or 600 mg) in the form of commercially available tablets, urine samples were collected just before, at least 1 h, and no later than 24 h after ingestion of IBU-containing pharmaceutical preparation as described above. All subjects involved in the study have also declared that, to the best of their knowledge, none of them suffer from any disease.

### Urine samples preparation

#### 2-HIBU and CIBU determination

The urine sample (100 µL) was mixed with 10 µL of 3 mol/L PCA. The mixture was shaken by hand for 1 min and then kept in a centrifuge at 12,000*g* for 5 min at 4 °C. A 5 µL aliquot of the upper layer of the resulting solution was assayed according to the procedure described in "2-HIBU and CIBU determination" section. Samples with concentrations that did not fall within the calibration range were diluted with ACN (the ratio of ACN to sample was predominantly 1:1) and re-assayed as described above.

#### Crn determination

Samples were prepared according to the slightly modified method of Kuśmierek et al.^30^. The urine sample (50 μL) was diluted by 500 times with deionized water. 10 µL of the resulting solution was injected into the HPLC system and assayed according to the procedure described in "Crn determination" section.

### Chromatographic conditions

#### 2-HIBU and CIBU determination

The IBU metabolites in urine samples, prepared according to the procedure described in "2-HIBU and CIBU determination" section, were separated using a mobile phase composed of 0.1% FA in water (solvent A) and ACN (solvent B) delivered at 1 mL/min the flow rate. The chromatographic separation was performed at 25 °C using the gradient elution: 0–4 min 30–45% B, 4–5 min 45–90% B, 5–6 min 90–30% B. The column was re-equilibrated between analyses by setting the post-run conditioning at 30% B for 2 min. The effluent was monitored with an ELSD detector operated with the following set of operation parameters: nebulizer temperature 90 °C, evaporator temperature 50 °C, gas (nitrogen) flow rate 1.3 SLM, data rate 10 Hz, photomultiplier tubes (PMT) gain of 10, smoothing 30 (3 s).

#### Crn determination

The chromatographic separation of the Crn in urine samples, prepared according to the procedure described in "Crn determination" section, was accomplished using the mobile phase consisting of water (solvent A) and MeOH (solvent B), delivered at the flow rate of 0.5 mL/min. The chromatographic separation was performed at 25 °C using gradient elution: 0–1 min 5% B, 1–2 min 5–30% B, 2–4 min 30–5% B, 4–8 min 5% B. The effluent was monitored with an ELSD detector operated with the following set of operation parameters: evaporator temperature 85 °C, nebulizer temperature 90 °C, gas (nitrogen) flow rate 1.15 SLM, data rate 10 Hz, PMT gain of 9, smoothing 30 (3 s).

### Institutional review board statement

The study was conducted according to the guidelines of the Declaration of Helsinki, and approved by the Bioethics Committee of the University of Lodz (decision identification code: 3/KBBN-UŁ/III/2020-21, date of approval 27.04.2021) as well as the Bioethics Committee of the Institute of the Polish Mother’s Memorial Hospital in Łódź (protocol code 35/2022, date of approval 12.04.2022).

### Informed consent

Written informed consent was obtained from all subjects involved in the study.

## Result and discussion

It is generally known that proper sample handling and management combined with separation and detection conditions play a pivotal role in the quality of generated results. In this study, experiments were carried out to provide the information regarding reliability of HPLC-ELSD assays. In particular, considerable attention was given to optimizing procedures and conditions related to selective separation and detection of the analytes. While designing of the methodology, a strong emphasis was put on greener analytical procedures considering each of the twelve principles of Green Analytical Chemistry (GAC)^31^.

During the study, the chemical and flow variables influencing each of the implemented sample preparation steps, and the chromatographic separation of analytes and their detection, were optimized in detail. All investigations were performed using the procedures described herein. In each case, the appearance of a particular analyte-delivered peak on the chromatogram and a comparison of its area/height were used to determine the process efficiency. All experiments concerning the optimization of sample preparation procedure as well as chromatographic and detection conditions were run at least in triplicate. The following (sub)sections of the article provide all necessary information regarding the development, validation, and in-study use of the described herein HPLC-ELSD based methods for the determination of primary metabolites of IBU, namely 2-HIBU and CIBU (method A) as well as Crn content (method B) in human urine.

### Sample preparation

Human urine primarily consists of 95% water, while the rest is urea (2%), Crn (0.1%), uric acid (0.03%), chloride, sodium, potassium, sulfate, ammonium, phosphate, and other ions and molecules in lesser amounts. Under normal conditions, protein is only found in trace amounts compared to their values in blood plasma^32–34^. In general, the urine of healthy individuals contains up to 150 mg of protein in the urine 24-h volume. In contrast, urinary protein excretion of more than 3.5 g per 24 h can occur in patients with proteinuria, according to the American Association for Clinical Chemistry. Since the HPLC-ELSD system cannot accommodate such kind of biomolecules, it has been concluded that sample deproteinization is needed in order to protect the analytical system against a decrease in its performance.

Urine samples were assayed for Crn according to a previously published procedure, based on HPLC–UV measurements^30^, involving sample dilution with deionized water. Importantly, this approach reduced the concentration of all interfering substances to an undetectable level. Regarding IBU metabolites assay, extensive sample dilution was excluded, taking into account the sensitivity of the elaborated method as well as the expected unknown 2-HIBU and CIBU concentration in study samples. In this case, sample preparation involved treatment with 3 mol/L PCA followed by centrifugation to remove urinary proteins. This is one of the most commonly used techniques for effectively eliminating proteins from biological samples, by the addition of water-miscible organic solvent and (ultra)filtration^35–38^. Based on our earlier findings, sample acidification with a popular protein precipitating agent, namely PCA, was taken into account. Importantly, the approaches utilizing centrifugal concentrators or membrane filters were excluded to minimize plastic consumption and reduce the quantity of the samples. The use of ACN, typically recognized as the most effective protein precipitating agent among organic solvents, was also eliminated due to a large excess of ACN needed in relation to biological sample, comparing to 3 mol/L PCA which is needed for complete removal of proteins^35–38^. Taking into account the trace concentration of urinary proteins, the efficient protein precipitation is achieved by mixing the urine sample with 3 mol/L PCA and crashing at 10:1 ratio by volumes. Since such an approach was beneficial for workflow simplification and results were satisfactory, no additional experimental work was undertaken to further optimize sample preparation step. Significantly, the duration and complexity of the sample pretreatment procedure was reduced in comparison with other methods^14–25^. The sample preparation time in our method only takes 5 min of centrifugation while in published methods, around 20 h^20,25^, 2.48 h (without evaporation to dryness)^17^, 2.05 h^23^, 2 h^14,15,18^, 46 min^19^, 10 min (without SPE)^21^. Longer sample preparation time is a consequence of using multi-step procedures involving SPE^15,16,20–22,25^, LLE^14,17,18,24^, solid phase microextraction^23^, USAEME^19^, hydrolysis^17,19–23,25^, evaporation to dryness^14,15,17,18,22,24^ and derivatization^14,15,17,18^.

Since chemical compounds can be decomposed prior to chromatographic analysis under different circumstances, the stability of the analytes under experimental conditions was evaluated. The problem has been approached qualitatively to measure the intactness of the analytes in a given matrix at room temperature for preselected time intervals. In the stability experiments, calibration standards at the lower/upper limit of quantification (L/U LOQ) were assayed according to the procedure of choice described in "Urine samples preparation" and "Chromatographic conditions" sections. Notably, it was found that Crn content remains stable for at least 7 days since the drop to 97.67% of the initial concentration is achieved when samples were left in the not temperature controlled autosampler. In parallel, there was no noticeable change in 2-HIBU and CIBU delivered peak area/height which remained stable for at least 1 working day at ambient temperature. Importantly, these results agree with the literature data and support information that Crn and IBU metabolites, namely 2-HIBU and CIBU in water (acidic) solutions, are stable^19,23,39,40^. In this way, sample handling and management effort can be significantly minimized due to the excellent stability of the analytes under experimental conditions. The analyte stability allows for preparation of a large batch of samples without the need of speed through the HPLC-ELSD method.

### Chromatographic and detection conditions

Among several separation techniques, HPLC has been the most frequently used technique to separate IBU, its metabolites, and Crn in complex systems^7–9,16,17,19,21,23,24^. Importantly, the above-mentioned compounds exhibit high compatibility with liquid phase separation techniques, due to their good solubility in commonly used mobile phases. In addition, these methods have taken advantage of separation in traditional reverse phase mode (RP-HPLC). In the present study, InfinityLab Poroshell 120 superficially porous column for RP-HPLC separations was chosen among those available in our laboratory. Importantly, we aimed to develop two different methods using the same analytical column in order to reduce expenses associated with running the analyses.

To successfully complete the project, a standard approach was employed to specify optimal conditions by assessing the influence of many operating parameters of the HPLC-ELSD system on the methods’ performance. Firstly, careful optimization of separation conditions was performed by taking into account operational guidelines provided by the column manufacturer. Many variables have been studied to find optimal conditions affording satisfactory separation performance. In principle, the best quality of results produced a satisfactory method selectivity by selecting the composition of the mobile phase (type of organic modifier, pH), its flow rate, and elution mode. As a result, crucial rules have been developed in the method to perform successful analysis. In particular, it was found that gradient elution was necessary to maintain the efficient resolution of the particular analyte peak from other sample components, properly equilibrate the chromatographic system between analyses, and reduce carry-over to the minimum level. Regarding Crn, it was also noted that an initial MeOH, instead of ACN, with a content no higher than 5%, and a flow rate of mobile phase set to 0.5 mL/min were essential to retain the analyte. In relation to IBU and its primary metabolites, successful separation of all sample constituents has been achieved using a typical mobile phase for HPLC-ELSD technique, consisting of ACN and water with 0.1% FA. Importantly, it has been found that separation had to be accomplished under acidic conditions in order to enhance the hydrophobicity of the target compounds to make them more compatible with stationary phase. Moreover, it has been recognized that the FA concentration, which was tested in the range of 0.1–1%, had a negligible effect on retention behaviors of solutes as well as resolution. Thus, ACN and water with 0.1% FA were selected as the eluent as they helped to decrease the noise level at high gain settings. Importantly, it has been recognized that the particular target compound peak produced a single symmetrical peak, which was well-resolved from other peaks on the column under set conditions. Therefore, no further experimental work was undertaken to optimize separation conditions during new methods development.

In the next stage of the method development process, ELSD detection conditions were optimized in order to increase sensitivity and selectivity in trace analysis. In the beginning, the evaporator temperature, nebulizer temperature, and gas flow rate parameters that greatly affects the detector sensitivity, were evaluated. Subsequently, frequency of data collection, applied signal amplification factor, and smoothing factor on signal quality were carefully studied. The initial experiments were conducted using the default settings of the ELSD detector. Then, each of the parameters mentioned above was adjusted to achieve the best performance. Importantly, only one parameter settings varied in the range specified by manufacturer with the constant value of other parameters. All experiments were run at least in triplicate, and the repeatability of the results, expressed as the coefficient of variation (CV) of peak height, was satisfactory. CV varied from 0.45 to 5.41% and 0.03 to 4.61% for Crn assay and IBU metabolites, respectively under any evaluated detection conditions. In general, predictable relationships of the data were obtained while optimizing the performance of the ELSD detector (Supplementary Materials, Figure S1 a–e and Figure S2 a–f). Finally, the specified operating conditions resulted in not only improvement of batch to batch reproducibility, but also sensitivity of the methods.

**Figure 1:**
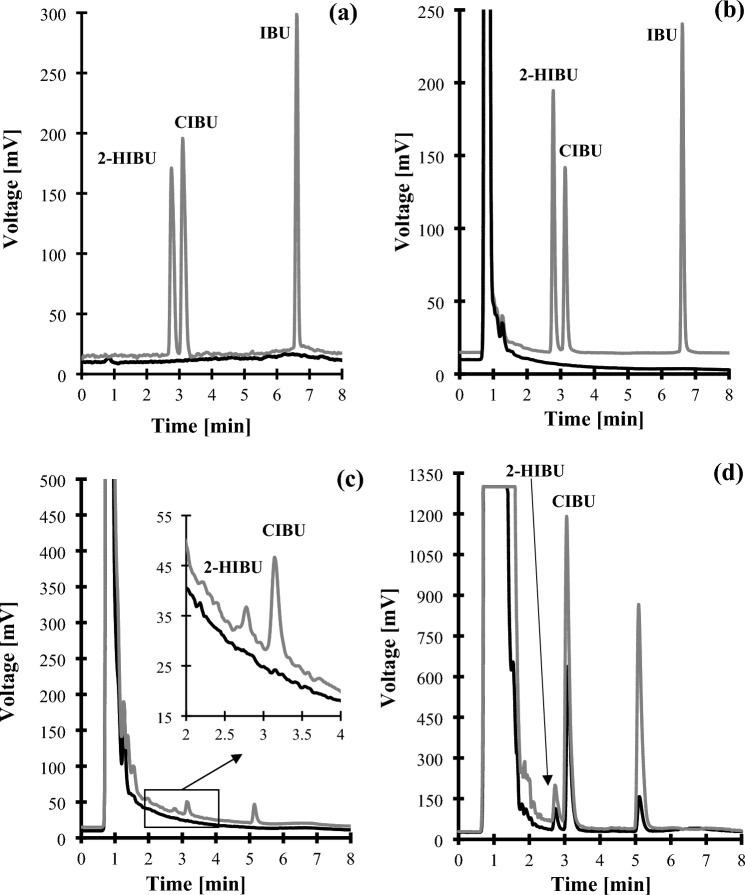
Representative chromatograms of standard solutions and human urine were prepared according to the procedure described in "2-HIBU and CIBU determination" section. Chromatographic conditions were as described in "2-HIBU and CIBU determination" section. (**a**) Blank standard solution (black line) and standard solution of IBU, 2-HIBU and CIBU (0.4 g/L in urine) (grey line); (**b**) normal human urine sample (black line) and the same sample spiked with the IBU, 2-HIBU and CIBU (0.4 g/L in urine) (grey line); (**c**) Normal human urine collected before (black line) and after 3 h oral ingestion of IBU-containing 400 mg pharmaceutical formulation (grey line); (**d**) Normal human urine collected after 3 h oral ingestion of IBU-containing 400 mg (black line) and 600 mg (grey line) pharmaceutical formulation (grey line). Under these conditions, the 2-HIBU, CIBU, and IBU peaks appear at 2.78 min, 3.13 min, and 6.61 min, respectively.

**Figure 2:**
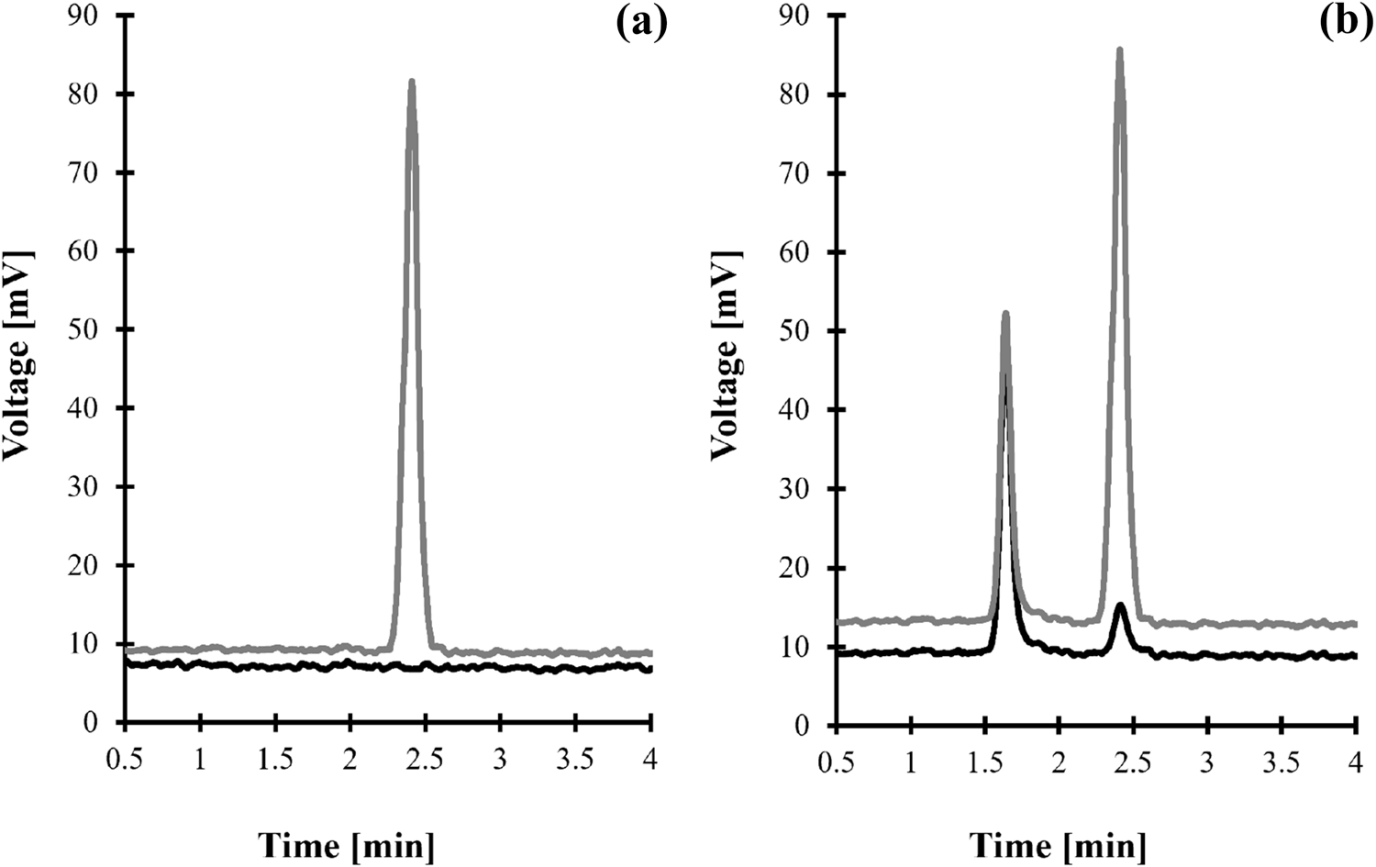
Representative chromatogram of standard solutions and human urine were prepared according to the procedure described in "Crn determination" section. Chromatographic conditions were as described in "Crn determination" section. (**a**) Blank standard solution (black line) and standard solution of Crn (15 mmol/L in urine) (grey line); (**b**) normal human urine sample (black line) and the same sample spiked with Crn (15 mmol/L in urine) (grey line). Under these conditions, the peak of Crn appears at 2.40 min.

Under optimized conditions described in "Chromatographic conditions" section, the 2-HIBU, CIBU, and Crn peak was approximately 65-times, 127-times, and 25-times higher in comparison with that registered with non-optimized ELSD settings, respectively. In addition, the peak of Crn (2.40 min), 2-HIBU (2.78 min), CIBU (3.13 min), and IBU (6.61 min) eluted within 8 min, and their corresponding peaks were easy to distinguish from the responses of all the concomitant components (Figs. 1b–d, 2b). Importantly, every time the elution profile of blank samples was free from any interference at the retention time of the analytes (Fig. 1a–c, 2a). The analysis time 8 min is more favorable than in other methods such as GC^14,15,18^, HPLC^16,17,21,23^ and CE^22,25^ where it is 17 min^14,18,22^, 21.8 min^15^, 30 min^23,25^, 35 min^16,21^, and 80 min^17^. The analysis time is the same as in CE method^20^ and worse than in HPLC method (1.8 min^19^ and 5 min^30^).

The identification and confirmation of the target compounds were performed by analyzing the standard solution of analytes processed according to the procedure described in "Urine samples preparation" and "Chromatographic conditions" sections. Each solution of a particular compound of interest was prepared separately and then was processed according to the method of choice to ascertain that a single analyte did not yield more than one chromatographic peak. In addition, the particular analyte peak was evaluated for purity by carrying out the analyses in a HPLC system coupled with both ELSD and UV detector. The UV detector was set to collect time and spectral information throughout the entire chromatogram. The spectra obtained during the elution of the target compound peak were compared. Importantly, the same spectra were acquired in different sections of the particular analyte peak, indicating its purity. Finally, the confirmation of the origin of each analyte peak and quantification of the compound of interest in real samples were based upon the comparison of retention time with the corresponding set of data obtained by analyzing an authentic compound.

### Greenness assessment of delivered HPLC-ELSD methods

In recent years, the assessment of an analytical procedure’s greenness is growing in popularity. There are several approaches which enable us to measure the degree of greenness of analytical methods. In the present study, AGREE—Analytical GREEnness metric approach and software (version 0.5 beta)^31,41–43^, which evaluates analytical procedures considering each of the twelve principles of GAC, was used to assess the greenness of the delivered HPLC-ELSD methods for urinary 2-HIBU and CIBU (method A) and Crn (method B) determination. Equal weights have been set for all twelve principles evaluated.

Regarding to the IBU metabolites assay, the following assumptions have been made in order to assess the analytical procedure’s greenness: the procedure involves an off-line analysis (principle 1); the volume of urine is 100 µL (principle 2); the analytical device is positioned off-line (principle 3); the number of distinctive analytical steps is two, including deproteinization combined with centrifugation, and chromatographic analysis (principle 4); the procedure is semi-automated and involves a miniaturized sample preparation methods (principle 5); derivatization step is not required (principle 6); the total amount of waste is 10.61 (g and mL combined), consisting of the sample itself, chemicals, and plastic disposable ware used to prepare the sample as well as mixture of solvents used during HPLC analysis (principle 7); two analytes are determined in a single run, and the sample throughput is seven samples per hour (principle 8); the most demanding technique is HPLC-ELSD (principle 9); none of the reagents are from renewable sources (principle 10), and finally the procedure requires no more than 3.5 mL of toxic chemical reagents (mobile phase ingredients) (principle 11) of which ACN is perceived as highly flammable and toxic to humans (principle 12). Since trace amounts of FA and PCA were used, they were not taken into account at the stage of assessing the operator`s safety.

**Figure 3:**
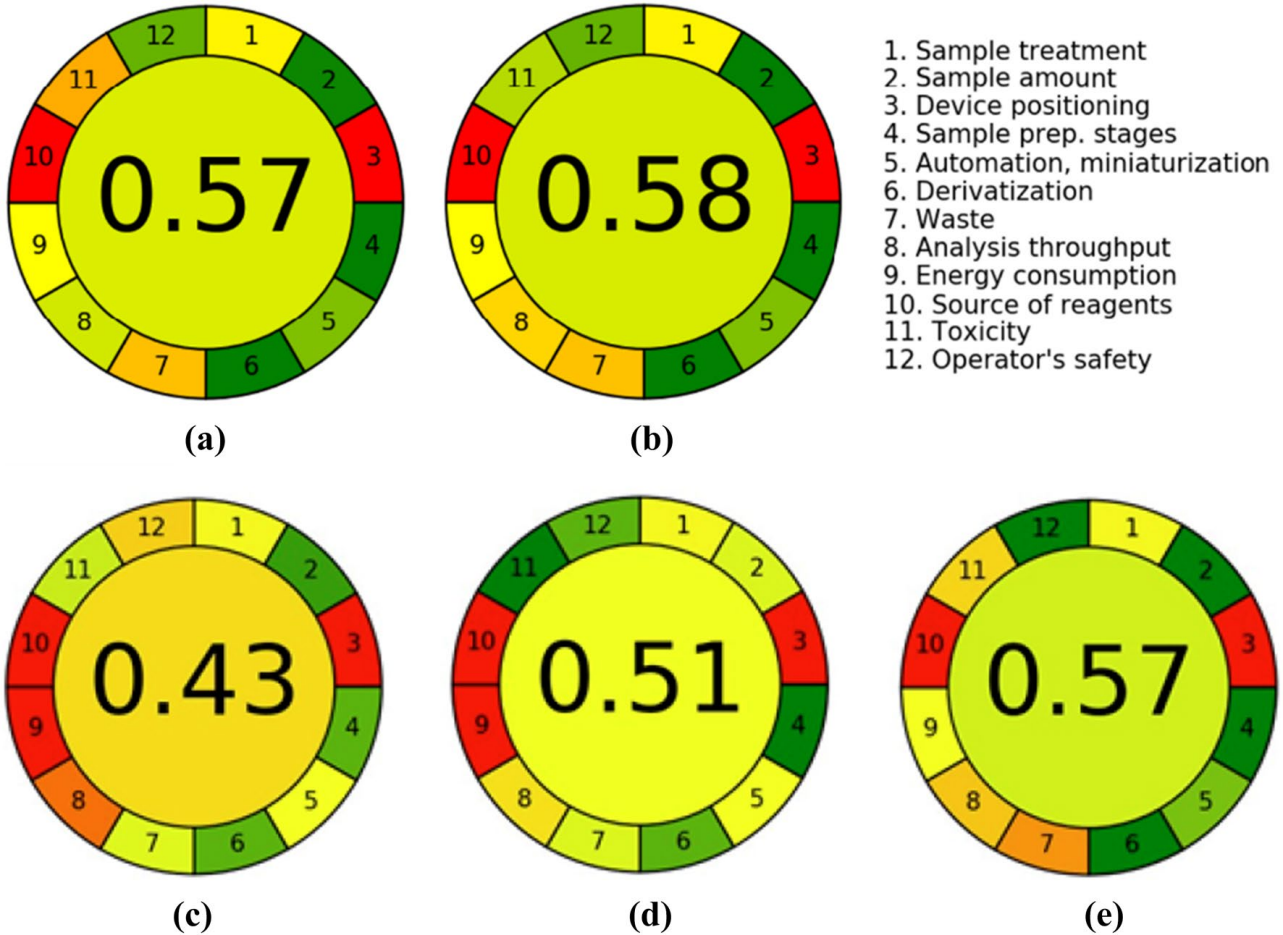
The assessment results with AGREE—Analytical GREEnness analysis of procedures for determination of (**a**) IBU metabolites, namely 2-HIBU and CIBU (method A), (**b**) Crn (method B) in human urine, (**c**) IBU metabolites in urine by GC–MS method^18^, (**d**) IBU metabolites in urine by HPLC–MS method^19^ and (**e**) Crn in urine by HPLC–UV method^30^.

In relation to Crn assay, it has been assumed that the procedure involves an off-line analysis (principle 1); the volume of urine is 50 µL (principle 2); the analytical device is positioned off-line (principle 3); the number of distinctive analytical steps is two, including sample dilution and HPLC-ELSD analysis (principle 4); the procedure is semi-automated and involves a miniaturized sample preparation methods (principle 5); Crn was not derivatized (principle 6); the total amount of waste is 10.25 (g and mL combined), consisting of the sample itself, chemicals, and plastic disposable ware used to prepare sample (principle 7); one analyte is determined in a single run, and the sample throughput is seven samples per hour (principle 8); the most demanding technique is HPLC-ELSD (principle 9); none of the reagents can be obtained from bio-based sources (principle 10), and finally the procedure requires approximately 0.35 mL of toxic chemical reagents (principle 11), while MeOH is perceived as highly flammable and toxic to humans (principle 12). The AGREE results for the methods under consideration are presented in Fig. 3.

Overall, it was concluded that the HPLC-ELSD assays fall into principles of GAC. The overall score, which is shown in the middle of the colored pictograms, is 0.57 and 0.58 in relation to IBU metabolites and Crn assays, respectively. Generally, values close to one and dark green in color indicate that the assessed procedure is greener. In addition, the use of AGREE—Analytical GREEnness metric approach helped identify the strong and weak points of our analytical procedures, whose distribution is comparable in both cases (Fig. 3). In particular, it has been assumed that the methods can be considered environmentally-friendly thanks to carrying out the chemical analysis on a very small scale combined with a low consumption of hazardous chemicals and laboratory disposable plastics. The assays have a relatively high-throughput potential and simplicity in sample preparation. Undoubtedly, a serious limitation of the presented methods is the use of energy-intensive measurement technique without the possibility of carrying out the analysis in situ.

We attempted to compare our methods in terms of environmental friendliness with other published assays for determination of IBU metabolites in urine by GC–MS^18^ and HPLC–MS^19^, and Crn by HPLC–UV^30^. Unfortunately, in these methods, the authors did not provide the degree of ecological friendliness of their procedures. Therefore, we estimated it using a dedicated calculator and data available in the articles. As can be seen in Fig. 3, our method for determining Crn (score 0.58) is comparable in terms of greenness to the HPLC–UV method (score 0.57)^30^, but for IBU metabolites (score 0.57) it is better than the GC–MS method (score 0.43)^18^ and HPLC–MS (score 0.51)^19^. This is due to the fact that the GC–MS and HPLC–MS techniques are more energy-consuming (principle 9) than HPLC-ELSD, which can be observed by comparing Fig. 3a with Fig. 3c, d. Moreover, both GC–MS and HPLC–MS methods have a much lower analysis throughput (principle 8) due to the long sample preparation time, requiring extraction in both methods and derivatization (90 min) in the GC–MS method^18^.

### Validation of the methods

Full validation of the new methods for determining 2-HIBU and CIBU (method A) and Crn (method B) concentration in human urine was conducted to establish that the performance characteristic of each procedure meets the requirements for the intended analytical application. All studies were planned and performed per the United States Food and Drug Administration (U.S. FDA) guidance for validation of bioanalytical methods^44^. Particularly, the process included evaluation of selectivity, linearity, intra-/inter-assay precision, accuracy, and LOQ. These essential parameters were measured in combined experiments. In addition, the matrix, dilution, and carry-over effects were evaluated during method validation. Some parameters were checked among system suitability testing. Detailed data regarding all evaluated validation parameters are presented herein.

#### System suitability

System suitability parameters, such as repeatability of chromatographic retention, expressed as CV of retention time, asymmetry factor, and number of theoretical plates, were selected during a particular validation method to determine instrument performance under optimized conditions. System suitability test calculations were performed as a part of linearity assessment by analyzing the calibration standards at the ULOQ in ten replicate injections. Good system suitability was demonstrated, ensuring that the system performs in an accurate and reproducible way. The CV value of retention time was 0.15%, 0.14% and 0.43% (acceptance criteria ≤ 1%), the mean asymmetry factor was 0.83, 0.81 and 0.99 (acceptance criteria 0.8–1.5), and number of theoretical plates was 6163, 7856 and 2818 (acceptance criteria ≥ 2000) for 2-HIBU, CIBU, and Crn, respectively.

#### Selectivity

The selectivity of a particular analyte in the presence of any other ELSD-producing signal endogenous components in a sample was verified during studies concerning the identification and confirmation of the origin of a particular analyte peak as described in "Chromatographic and detection conditions" section. Crn selectivity studies also assessed interferences originating from urea as it is the most abundant component of urine apart from water^32–34^. The blank standard solution (water) and standard solution of urea were assayed according to the procedure described in "Crn determination" and "Crn determination" sections. As shown in Fig. 2a, the elution profile is free from any interferences at the Crn peak's retention time. Under these conditions, urea is not retained, and its corresponding peak is eluted before the column dead volume. To confirm the finding, normal urine samples from six individual sources and the same samples spiked with urea were assayed according to the procedures described herein ("Crn determination" and "Crn determination" sections). No increase in the peak area/height of Crn was observed.

Regarding 2-HIBU and CIBU, the selectivity studies evaluated interferences originating from all (un)known endogenous components of urine and the precursor of metabolites mentioned above, namely IBU. Firstly, a blank standard solution [a mixture of MeOH in water (50:50, *v/v*)] and a standard solution of IBU were prepared and analyzed according to the procedure described in "2-HIBU and CIBU determination"and "2-HIBU and CIBU determination" sections, respectively. Normal urine samples from six control subjects and the same samples spiked with IBU were assayed according to the procedures described herein. As shown in Fig. 1a–c, no response attributable to interfering components was observed at the retention time of 2-HIBU and CIBU, denoting the ability of HPLC-ELSD method to differentiate and measure the analytes in the presence of interfering substances in urine samples.

#### Linearity

An external standard calibration method was used to verify the calibration range of the methods. For this purpose, the multilevel calibration curves consisted of a blank sample, and six calibrators, including LOQ, were generated for each analyte and were run in triplicate over 3 subsequent working days. Each set contained calibration standards at the level of 0.5, 1, 5, 10, 15, 30 mmol/L regarding Crn assay, and 0.06; 0.1; 0.2; 0.3; 0.4; 0.5 g/L concerning 2-HIBU and CIBU assay. Importantly, the calibration curve for Crn consisted of points that cover the entire range of expected analyte concentrations in the test samples. In contrast, the relatively narrow calibration range of the IBU metabolites method was experimentally defined by assessing the lowest and highest calibration standards whose nominal 2-HIBU and CIBU concentration and the response of the analytical platform to the particular analyte were well fitted as described below. Significantly, such an approach has been employed to evaluate the calibration range of the IBU metabolites assay. It is almost impossible to specify the entire expected analytes concentration range in the test urine samples which are highly dependent on the dose of the IBU-containing painkillers. Calibration standards were prepared in our laboratory pooled urine by spiking with known quantities of the particular analyte. Since urine samples free of Crn were unavailable, the endogenous concentration of the analyte was evaluated before the calibration curves preparation by triplicate analysis.

The linearity was initially evaluated graphically by visually inspecting a plot of the peak area/height as a function of the particular analyte concentration and by using the least-squares regression model to describe the concentration–response relationship. Since it has been recognized that ELSD response increased with an increase in any analyte concentration in a non-linear manner, a few calibration curve fitting methods were tested to fit experimental data to a linear calibration curve. Overall, a very good linear fit of log ELSD response against log analyte concentration was observed in the case of each method. In addition, the calibration standards fitted well into the linear regime, which gave correlation coefficient values of at least 0.9970, showing that the instrument response was proportional to the analytes’ concentration within the experimentally defined quantitation range. Data dealing with validation parameters, evaluated as a part of the linearity assessment using an external standard calibration method, and calculated from the peak height values, are shown in Table 1.

**Table 1:** Validation data corresponding to intra-assay measurements (n = 3).

Analyte	Regression equation	R	Linear range (mmol/L)^a^ (g/L)^b^	Intra-assay precision (%)		Intra-assay accuracy (%)		LOQ (mmol/L)^a^ (g/L)^b^
				Min	Max	Min	Max	
2-HIBU	y = 1.687x + 0.084	0.9980	0.06–0.5^b^	1.06	14.83	85.10	105.92	0.06^b^
CIBU	y = 1.757x + 0.091	0.9970	0.06–0.5^b^	1.50	11.51	89.00	103.33	0.06^b^
Crn	y = 1.215x + 0.921	0.9983	0.5–30^a^	0.67	4.29	81.19	114.72	0.5^a^

*CIBU* carboxyibuprofen, *Crn* creatinine, *LOQ* limit of quantification, *R* correlation coefficient, *2-HIBU* 2-hydroxyibuprofen.

Conducted experiments also indicated that only the particular analyte-delivered signal peak area/height increased with its growing concentration. Substantial changes in the slope of any regression line obtained across the day, as well as over 3 subsequent days were not observed, suggesting that the presented analytical methods are not affected by matrix components.

#### Accuracy and precision

The accuracy and precision of the assay, referring to intra- and inter-day measurements, were evaluated as a part of a linearity assessment. The precision was expressed as the CV of measurement repeatability, whereas accuracy was the percentage of analyte recovery. In particular, accuracy was calculated by expressing the mean measured amount as a percentage of added amount of a particular analyte. In the Crn assay, as the analyte is also an endogenous molecule, the concentration of the endogenous molecule in the blank matrix was determined and subtracted from the total concentrations observed in the spiked samples. As recommended by U.S. FDA^44^, the accuracy of the Crn assay was explicitly calculated using of the following formula *Accuracy (%)* = *[(measured amount* − *endogenous content)/added amount]* × *100*. Intra-assay precision and accuracy were demonstrated by triplicate analysis of freshly prepared calibrators, which referred to pooled urine samples containing known amounts of the particular analyte at three different levels corresponding to values close to LLOQ, the middle of the quantitation range, and ULOQ, respectively. Experiments concerning estimating the intermediate accuracy and precision were repeated in the same manner over 3 subsequent days. All concentrations were tested using the calibration curves prepared on that occasion. Importantly, the obtained results from analytical runs met the acceptance criteria. In relation to Crn, the accuracy ranged from 81.19 to 114.72% and 81.19 to 108.72% for intra- and inter-day variation, respectively. In parallel, the precision varied from 0.67 to 4.29% and 2.20 to 13.15% for intra- and inter-day measurements, respectively. Regarding IBU metabolites, the precision did not exceed 14.83% of CV at any examined concentration level. It varied from 1.06 to 14.83% and 6.39 to 14.53% for intra- and inter-day measurements, respectively. In parallel, accuracy ranged from 85.10 to 105.92% and 86.96 to 108.08% for intra- and inter-day variation, respectively. Detailed data on precision and accuracy from the 3 day experiments, compared with intra-assay precision and accuracy, are shown in Table 2.

**Table 2:** Precision and accuracy of the data (n = 3).

Analyte	Concentration (mmol/L)^a^ (g/L)^b^	Precision (%)		Accuracy (%)	
		Intra-assay	Intermediate	Intra-assay	Intermediate
2-HIBU	0.06^b^	2.54	11.16	85.10	86.96
	0.3^b^	10.04	14.53	103.49	103.79
	0.5^b^	1.06	13.81	105.92	108.08
CIBU	0.06^b^	2.50	8.95	89.00	94.51
	0.3^b^	8.24	14.01	102.08	101.23
	0.5^b^	1.50	9.40	103.33	101.75
Crn	0.5^a^	3.60	8.85	81.19	91.10
	10^a^	3.39	13.15	108.72	94.45
	30^a^	4.29	2.20	99.49	101.16

*CIBU* carboxyibuprofen, *Crn* creatinine, *2-HIBU* 2-hydroxyibuprofen.

#### The limit of quantification

LOQ was evaluated in parallel with the calibration range’s intra-assay precision and accuracy assessment. The LLOQs, are equal to 0.06 g/L and 0.5 mmol/L in urine for the IBU metabolites and Crn, respectively, were accepted as LOQ. In each case, this concentration of the particular analyte produced easy to distinguish from the background noise and reproducible detector response with a precision that did not exceed 11.16%, and accuracy ranged from 81.19 to 120.16%. In addition, the estimated LOQ values closely corresponded to the LOQs determined experimentally by the signal-to-noise method. In this method, a blank urine sample or a surrogate matrix (water) was enriched with decreasing concentrations of the particular analyte regarding IBU metabolites and Crn, respectively. The surrogate matrix was used to evaluate the LOQ of Crn assay because obtain a urine sample free from Crn was impossible. Samples were then handled according to the procedures described in "Urine samples preparation" and "Chromatographic conditions" sections until the injected amount resulted in a peak 10 times as high as the baseline signal. Notable, the obtained LOQ values were satisfactory and allowed the determination of the compounds of interest content in human urine.

#### Matrix effect

The effect of the matrix between different independent sources, defined as an alteration in analyte(s) response due to interfering and usually unidentified sample components, was assessed in a relevant volunteer population. The volunteer population refers to control subjects who were apparently healthy volunteers involved in the study. Apart from selectivity and dilution integrity studies, the matrix effect assessment involved comparing calibration curves of the six individual sources of urine samples against a calibration curve of the pooled matrix and surrogate matrix (water). Importantly, it was recognized that the CV of the slope of the regression lines did not deviate by more than 3.44%, 2.71%, and 0.86% in relation to Crn, 2-HIBU, and CIBU, respectively, denoting the absence of any matrix effect. In fact, with this difference in slopes, the maximum error in analytical results of Crn, 2-HIBU, and CIBU would be 8.74%, 5.20%, and 1.46% respectively. Thus, an external standard addition method was used to establish the levels of the particular analyte in urine samples as it provides the procedure’s reliability along with effort minimization.

#### Dilution integrity

The dilution integrity of IBU metabolites has been assessed as the method measures diluted samples. In order to evaluate the impact of the sample dilution procedure on the measured concentration of the analyte, calibration standards at the concentration above the ULOQ (0.8 g/L in urine) were prepared in urine from six individual sources and the surrogate matrix. The samples were diluted with ACN and analyzed according to the procedure described in "2-HIBU and CIBU determination"and "2-HIBU and CIBU determination" sections. Five different dilution factors, corresponding to the expected dilutions in the study, were evaluated that amounted to 1–5 times the volume of the (urine) sample. Notably, the mean accuracy and precision of these diluted calibration standards were 96.30% and 9.56%, respectively. In parallel, it has been recognized that instrument response was directly proportional to the 2-HIBU, and CIBU concentration and the CV of the slope of the regression lines did not deviate by more than 3.44% concerning any matrix under consideration. In addition, 2-HIBU and CIBU detector signals of the same peak height/area were registered regardless of the matrix type. Importantly, these results have proved that the measured concentrations are not affected by the magnitude by which the samples were diluted within the calibration range. Along with the documented high accuracy and precision, this demonstrates that the method can analyze samples at a concentration exceeding the ULOQ of the calibration curve without the influence of a matrix.

#### Carry-over

Carry-over between samples, meaning the appearance of an analyte in a sample from a preceding sample, can occur in analytical methods. As it may impact on the precision and accuracy of the study sample concentrations, the potential of carry-over was thus investigated in the study as a part of the linearity assessment. In each case, the standard blank solution sample(s) were placed after the calibration standard at the ULOQ to evaluate the carry-over. Importantly, each time the response of blank samples was as high as the background signal, indicating that the carry-over effect did not occur. The registered chromatograms were similar to the elution profile of blank samples presented in Figs. 1a–c and 2a.

In conclusion, it has been shown that upon validation the performance of the presented methods is suited to the analysis of study samples. Importantly, it has been demonstrated that the methods are sensitive enough and have suitable precision, accuracy, and linearity levels, falling within acceptable tolerance limits^44^. Importantly, it has been recognized throughout the application of the methods that carry-over between samples did not occur in any analytical method and matrix as well as sample dilution have a negligible impact on these assays results. Based on the analysis of validation data, it has been concluded that the performance of the presented HPLC-ELSD based methods is sufficient to allow them to be used in diagnostic testing.

### Application of the methods

The validated HPLC-ELSD assays were used to quantitatively determine IBU metabolites, namely 2-HIBU, CIBU, and Crn in urine samples from apparently healthy human subjects. Since it has been recognized that the assays are not affected by matrix components, an external standard addition method was used to establish urinary levels of the analytes in study samples, handled according to the procedures described in "Urine samples preparation" and "Chromatographic conditions" sections. Importantly, 2-HIBU and CIBU were only detected in study samples delivered by donors who have ingested orally IBU-containing pharmaceutical preparation, while Crn was found in all urine samples. The concentration of the analytes in each sample was calculated using the mathematical formula corresponding to the equation of the calibration line generated on that occasion. Importantly, the results for urinary 2-HIBU and CIBU content were adjusted for Crn in order to facilitate comparison of different individuals. The estimated concentrations of urinary 2-HIBU, CIBU, and Crn, based on data obtained by triplicate analysis of a particular sample from an individual source, varied from 0 to 0.266 g/L (0–34.68 g/mol Crn), from 0 to 0.495 g/L (0–77.68 g/mol Crn) and from 0.64 to 23.88 mmol/L in urine respectively. Importantly, these values were similar to those previously reported, using different technical approaches^7–9,16,17,19–25^, denoting the data’s reliability from the presented HPLC-ELSD assays.

**Figure 4:**
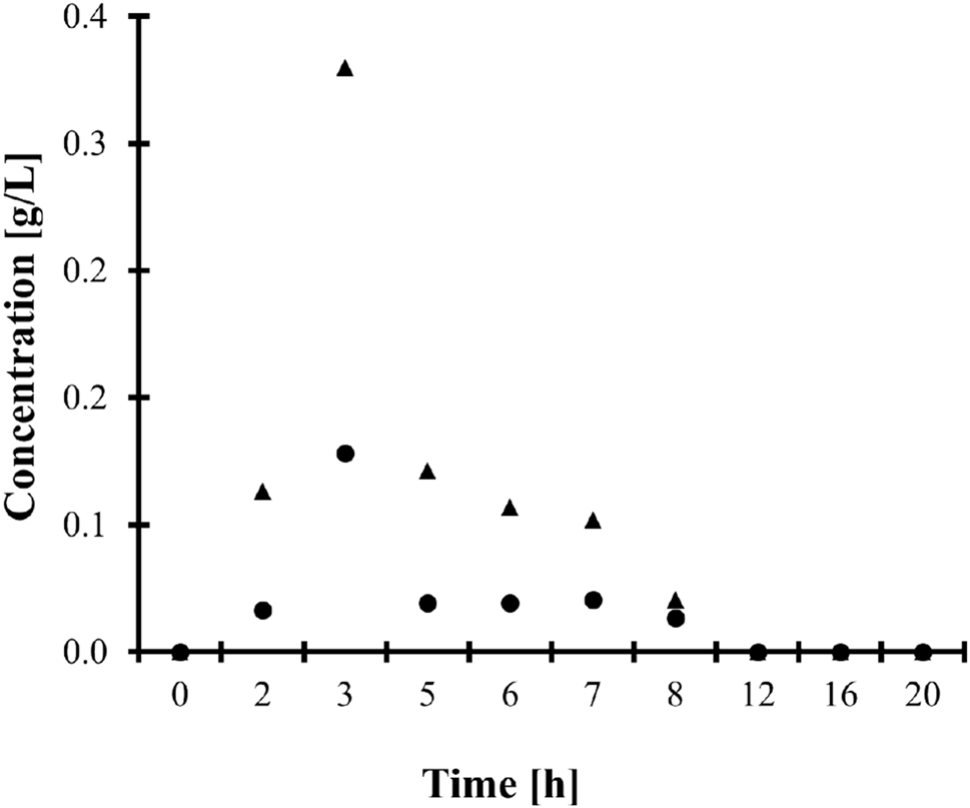
Renal excretion of IBU metabolites, namely 2-HIBU (dots) and CIBU (triangles), after oral administration of one dose of IBU-containing 400 mg tablets.

In addition, the HPLC-ELSD assay was used to perform clinical pharmacokinetic studies of IBU. One apparently healthy adult volunteer was involved in the experiment. The donor has administered one dose of IBU-containing 400 mg tablet orally, which did not exceed the recommended drug dose. Urine samples were collected just before and several times within 24 h after ingestion of the drug as described in "Biological samples collection" section, and then handled according to the procedure described in "Urine samples preparation" and "Chromatographic conditions" sections. First, these in vivo experiments have confirmed the identity of the peaks eluting at 2.78 min and 3.13 min indicating that they are derived from 2-HIBU and CIBU present in samples after urine donors ingested a drug orally (Fig. 1c). Moreover, it has been recognized that IBU is excreted by the kidney within no later than 24 h after ingestion of the drug (Fig. 4), and the concentration of CIBU was higher than the concentration of 2-HIBU (Fig. 1d), in agreement with literature data^1,2^. Moreover, it has been demonstrated that the presented HPLC-ELSD assay is suitable for screening human urine in terms of drug metabolites.

## Conclusions

To the best of our knowledge, the article presents the first HPLC-ELSD based methods for simultaneous assessment of IBU and its main metabolites (2-HIBU and CIBU) and Crn content in human urine. In particular, the attractiveness of the presented HPLC-ELSD assays relies on (1) a streamlined one-step sample preparation procedure followed by (2) a short chromatographic analysis as well as (3) a possibility of carrying out chemical analysis on a small scale combined with (4) low consumption of hazardous chemicals and laboratory disposable plastic, and (5) greater greenness resulting using less energy than HPLC–MS and GC–MS methods. For instance, using the presented assays for preparing of a set of twenty four samples and their HPLC analysis for IBU metabolites and Crn takes no more than 45 min and 6.5 h, respectively, considering all the operations that need to be performed. This achievement in terms of the sample preparation step is the same for Crn (45 min) compared to HPLC–UV method^30^ and is better for IBU metabolites compared to GC–MS^14,15,18^ and HPLC–MS^19^ methods, where this process takes 2.5 h and 53 min, respectively, for twenty four samples. However, the time of HPLC analysis of the same number of samples is better or similar to GC–MS methods (8.72 h^15^ and 6.84 h^14,18^), and worse than HPLC–MS method (43.2 min^19^). In addition, sample handling and preparation is accompanied by consumption of as little as 0.01 mL 3 mol/L PCA and approximately 2.40 mL deionized water per one sample, which represent inexpensive and non-toxic chemicals. In contrast, the GC–MS^14,18^ and HPLC–MS^19^ methods use greater volumes of additives such as diethyl ether (2 mL), ethyl acetate (0.1 mL), methyl iodide (0.05 mL), and anhydrous potassium carbonate (50 mg) in GC–MS method, and 1 mol/L sodium hydroxide (0.4 mL), 1 mol/L hydrochloric acid (0.4 mL), 1-octanol (0.1 mL), and MeOH (0.09 mL) in HPLC–MS method. Moreover, the semi-automation of the analytical procedures brought beneficial consequences as it reduces labor intensity, maximizes sample throughput and improves the accuracy and reproducibility of the methods. Interestingly, presented assays have produced compelling evidence supporting the conclusion that 2-HIBU and CIBU are main metabolites of IBU, as reported elsewhere^1,2^. Importantly, the HPLC-ELSD based methods provide new analytical tools that can facilitate studies of the compounds mentioned above in health and disease, for example, by providing information about acute overdose or chronic abuse of IBU-containing drugs. In our opinion, these methods are free of restrictions. It nonetheless needs to be emphasized that a successful analysis using the proposed methods can only be achieved when the recommended sample handling and management procedures are followed.

### Supplementary Information

Supplementary Figures.

## Data Availability

Essential data is contained within the article and its Supplementary Materials file. In addition, the dataset generated and analyzed during this study, which contributed to the article, can be made available by the corresponding authors (J.P. and G.C.) upon reasonable request as long as the request does not compromise intellectual property interests. Urine samples are not available from the authors.

## Abbreviations

ACN: Acetonitrile
CE: Capillary electrophoresis
CIBU: Carboxyibuprofen
Crn: Creatinine
CV: Coefficient of variation
ELSD: Evaporative light scattering detection
FA: Formic acid
GAC: Green Analytical Chemistry
GC: Gas chromatography
IBU: Ibuprofen
LLE: Liquid–liquid extraction
(L/U) LOQ: (Lower/upper) limit of quantification
MeOH: Methanol
MS: Mass spectrometry
PCA: Perchloric acid
PMT: Photomultiplier tubes
(RP)-HPLC: (Reverse-phase) liquid chromatography
SPE: Solid phase extraction
U.S. FDA: United States Food and Drug Administration
USAEME: Ultrasound-assisted emulsification microextraction
UV: Ultraviolet detection
2-HIBU: 2-Hydroxyibuprofen

## References

[1] *Ibuprofen: Discovery, Development and Therapeutics* (Wiley, 2015). 10.1002/9781118743614.

[2] Rainsford KD (2013). Ibuprofen: Pharmacology, Therapeutics and Side Effects.

[3] Ibuprofen. in *Meyler’s Side Effects of Drugs. The International Encyclopedia of Adverse Drug Reactions and Interactions* (ed. Aronson, J. K.) (Elsevier, 2016). 10.1016/B978-0-444-53717-1.00873-8.

[4] Ibuprofen. in *Encyclopedia of Toxicology* (ed. Wexler, P.) (Academic Press, 2014). 10.1016/B978-0-12-386454-3.00739-9.

[5] González-Domínguez R, González-Domínguez Á, Sayago A, Fernández-Recamales Á (2020). Recommendations and best practices for standardizing the pre-analytical processing of blood and urine samples in metabolomics. Metabolites.

[6] Wu Y, Li L (2016). Sample normalization methods in quantitative metabolomics. J. Chromatogr. A.

[7] Narimani R, Esmaeili M, Rasta SH, Khosroshahi HT, Mobed A (2021). Trend in creatinine determining methods: Conventional methods to molecular-based methods. Anal. Sci. Adv..

[8] Pundir CS, Kumar P, Jaiwal R (2019). Biosensing methods for determination of creatinine: A review. Biosens. Bioelectron..

[9] Jayasekhar Babu P (2022). Conventional and nanotechnology based sensors for creatinine (A kidney biomarker) detection: A consolidated review. Anal. Biochem..

[10] Řemínek R, Foret F (2021). Capillary electrophoretic methods for quality control analyses of pharmaceuticals: A review. Electrophoresis.

[11] Petrie B, McAdam EJ, Scrimshaw MD, Lester JN, Cartmell E (2013). Fate of drugs during wastewater treatment. TrAC Trends Anal. Chem..

[12] Carlucci G, Carlucci M, Locatelli M (2012). Analysis of anti-inflammatory enantiomers by HPLC in human plasma and urine: A review. Antiinflamm. Antiallergy Agents Med. Chem..

[13] Buchberger W (2011). Current approaches to trace analysis of pharmaceuticals and personal care products in the environment. J. Chromatogr. A.

[14] Waraksa E (2019). Quantification of unconjugated and total ibuprofen and its metabolites in equine urine samples by gas chromatography–tandem mass spectrometry: Application to the excretion study. Microchem. J..

[15] Waraksa E (2018). Simultaneous determination of ibuprofen and its metabolites in complex equine urine matrices by GC-EI-MS in excretion study in view of doping control. J. Pharm. Biomed. Anal..

[16] Kepp DR, Sidelmann UG, Tjornelund J, Hansen SH (1997). Simultaneous metabolites liquid quantitative determination of the major phase I and II of ibuprofen in biological fluids by high-performance chromatography on dynamically modified silica. J. Chromatogr. B.

[17] Rudy AC, Anliker KS, Hall SD (1990). High-performance liquid chromatographic determination of the stereoisomeric metabolites of ibuprofen. J. Chromatogr. B Biomed. Sci. Appl..

[18] Waraksa E (2018). A rapid and sensitive method for the quantitative analysis of ibuprofen and its metabolites in equine urine samples by gas chromatography with tandem mass spectrometry. J. Sep. Sci..

[19] Magiera S, Gülmez Ş (2014). Ultrasound-assisted emulsification microextraction combined with ultra-high performance liquid chromatography-tandem mass spectrometry for the analysis of ibuprofen and its metabolites in human urine. J. Pharm. Biomed. Anal..

[20] Karaźniewicz-Łada M, Łuczak M, Główka F (2009). Pharmacokinetic studies of enantiomers of ibuprofen and its chiral metabolites in humans with different variants of genes coding CYP2C8 and CYP2C9 isoenzymes. Xenobiotica.

[21] Djukovic D (2008). Ibuprofen metabolite profiling using a combination of SPE/column-trapping and HPLC-micro-coil NMR. J. Pharm. Biomed. Anal..

[22] Główka F, Karaźniewicz M (2007). Enantioselective CE method for pharmacokinetic studies on ibuprofen and its chiral metabolites with reference to genetic polymorphism. Electrophoresis.

[23] De Oliveira ARM, De Santana FJM, Bonato PS (2005). Stereoselective determination of the major ibuprofen metabolites in human urine by off-line coupling solid-phase microextraction and high-performance liquid chromatography. Anal. Chim. Acta.

[24] Tan SC, Patel BK, Jackson SHD, Swift CG, Hutt AJ (2002). Stereoselectivity of ibuprofen metabolism and pharmacokinetics following the administration of the racemate to healthy volunteers. Xenobiotica.

[25] Bjørnsdottir I, Kepp DR, Tjørnelund J, Hansen SH (1998). Separation of the enantiomers of ibuprofen and its major phase I metabolites in urine using capillary electrophoresis. Electrophoresis.

[26] Magnusson LE, Risley DS, Koropchak JA (2015). Aerosol-based detectors for liquid chromatography. J. Chromatogr. A.

[27] Mojsiewicz-Pieńkowska K (2009). On the issue of characteristic evaporative light scattering detector response. Crit. Rev. Anal. Chem..

[28] Lucena R, Cárdenas S, Valcárcel M (2007). Evaporative light scattering detection: Trends in its analytical uses. Anal. Bioanal. Chem..

[29] Megoulas NC, Koupparis MA (2005). Twenty years of evaporative light scattering detection. Crit. Rev. Anal. Chem..

[30] Kuśmierek K, Głowacki R, Bald E (2006). Analysis of urine for cysteine, cysteinylglycine, and homocysteine by high-performance liquid chromatography. Anal. Bioanal. Chem..

[31] Tobiszewski M, Mechlińska A, Namieśnik J (2010). Green analytical chemistry—Theory and practice. Chem. Soc. Rev..

[32] Sarigul N, Korkmaz F, Kurultak İ (2019). A new artificial urine protocol to better imitate human urine. Sci. Rep..

[33] Aitekenov S, Gaipov A, Bukasov R (2021). Review: Detection and quantification of proteins in human urine. Talanta.

[34] Turitto V, Slack S, Murphy W, Black J, Hastings G (2016). Blood and related fluids. Handbook of Biomaterial Properties.

[35] Daykin CA, Foxall PJD, Connor SC, Lindon JC, Nicholson JK (2002). The comparison of plasma deproteinization methods for the detection of low-molecular-weight metabolites by 1H nuclear magnetic resonance spectroscopy. Anal. Biochem..

[36] Piechocka J, Głowacki R (2023). Up-to-date knowledge about analytical methods for homocysteine thiolactone determination in biological samples. TrAC Trends Anal. Chem..

[37] Głowacki R, Piechocka J, Bald E, Chwatko G, Buszewski B, Baranowska I (2022). Application of separation techniques in analytics of biologically relevant sulfur compounds. Handbook of Bioanalytics.

[38] Piechocka J, Wrońska M, Głowacki R (2020). Chromatographic strategies for the determination of aminothiols in human saliva. Trends Anal. Chem..

[39] Spierto FW, Hannon WH, Gunter EW, Smith SJ (1997). Stability of urine creatinine. Clin. Chim. Acta.

[40] Miki K, Sudo A (1998). Effect of urine pH, storage time, and temperature on stability of catecholamines, cortisol, and creatinine. Clin. Chem..

[41] Pena-Pereira F, Wojnowski W, Tobiszewski M (2020). AGREE—Analytical GREEnness metric approach and software. Anal. Chem..

[42] Pena-Pereira F, Tobiszewski M, Wojnowski W, Psillakis E (2022). A tutorial on AGREEprep an analytical greenness metric for sample preparation. Adv. Sample Prep..

[43] Wojnowski W, Tobiszewski M, Pena-Pereira F, Psillakis E (2022). AGREEprep—Analytical greenness metric for sample preparation. TrAC Trends Anal. Chem..

[44] FDA. *Bioanalytical Method Validation Guidance for Industry* (FDA, Rockville, MD, USA, 2018).

