# Supplementary material for: Application of the HPLC-ELSD technique for the determination of major metabolites of ibuprofen and creatinine in human urine
Source: Sci Rep. 2023 Nov 20;13:20268. doi: 10.1038/s41598-023-47594-8 (PMC10662266; doi:10.1038/s41598-023-47594-8)
Supplement: Supplementary file 1 — Supplementary Figures. [file 41598_2023_47594_MOESM1_ESM.docx]

**Application of the HPLC-ELSD technique for the determination of major metabolites of ibuprofen and creatinine in human urine**

Justyna Piechocka^1^*, Natalia Matwiej^1^, Marta Gaweł^1,2^, Michał Matyjaszczyk^3,4^,
Rafał Głowacki^1^, Grażyna Chwatko^1^*

*^1^ University of Lodz, Faculty of Chemistry, Department of Environmental Chemistry, Pomorska 163/165, 90-236, Łódź, Poland*

*^2^ Doctoral School of Exact and Natural Sciences, University of Lodz, Banacha 12/16,
90-237 Łódź, Poland*

*^3^ Polish Mother's Memorial Hospital Research Institute, Department of Family Medicine, Rzgowska 281/289, 93-338 Łódź, Poland*

*^4^ Medical University of Lodz, Department of Family Medicine, Narutowicza 60,
90-131 Łódź, Poland*

**Corresponding author(s)**

* Department of Environmental Chemistry, Faculty of Chemistry, University of Lodz, 163/165 Pomorska, 90-236 Łódź, Poland

J.P.: e-mail: justyna.piechocka@chemia.uni.lodz.pl; Tel.:+48-42-635 5844

G.C.: e-mail: grazyna.chwatko@chemia.uni.lodz.pl; Tel.:+48-42-635 5843

**Supplementary results produced during optimization of ELSD detection conditions**

**Figures and tables**

**Figure S1.**

**
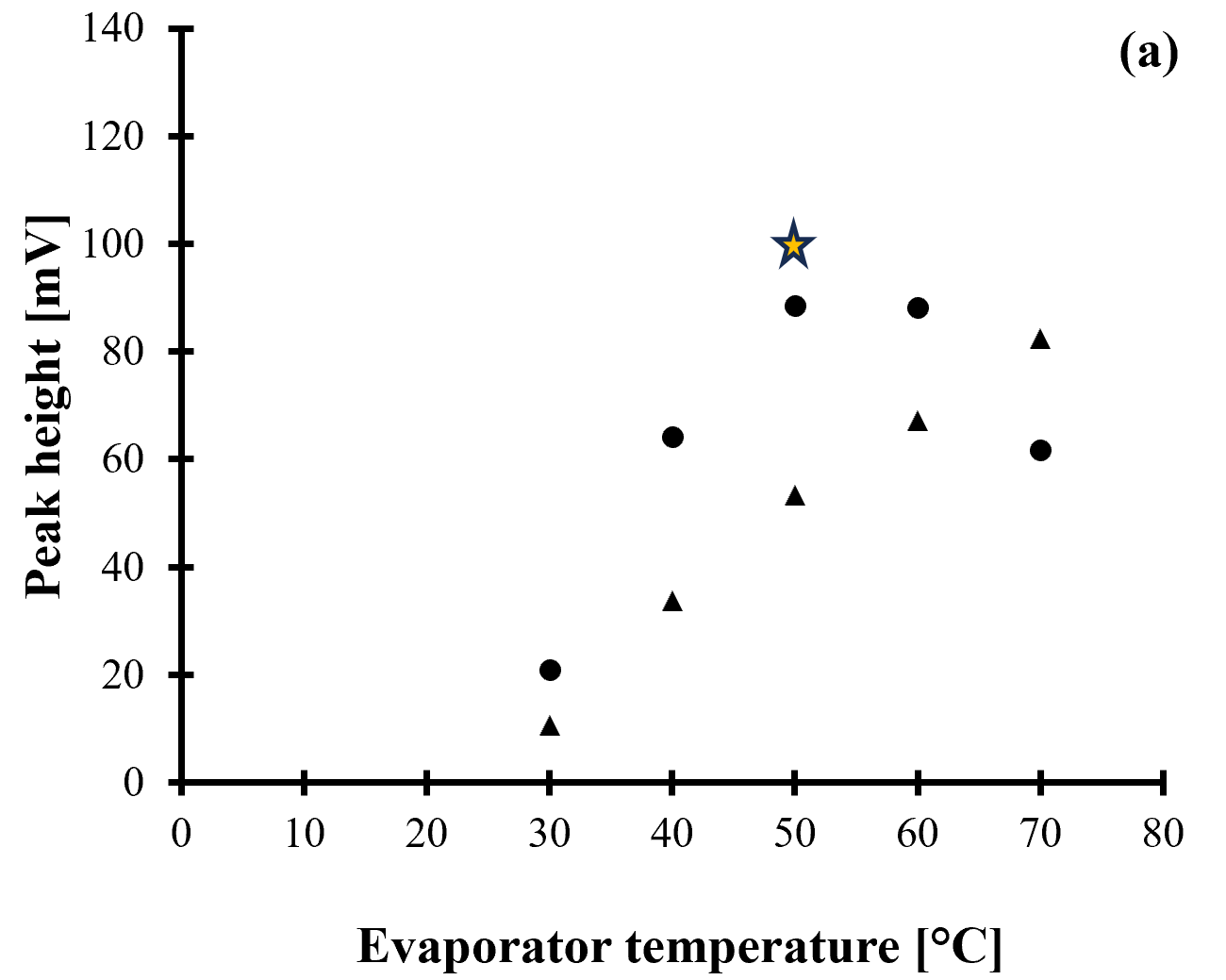

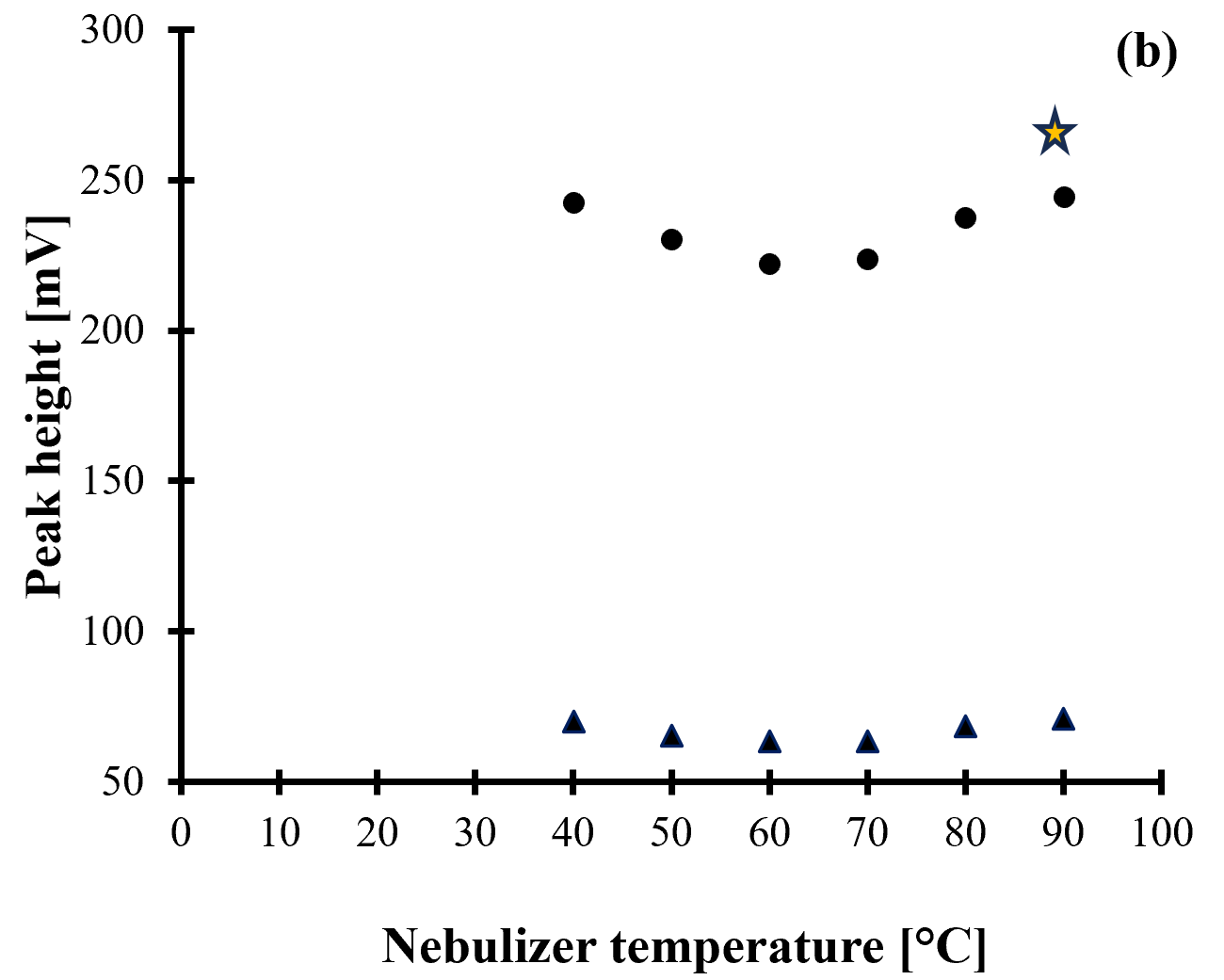
**

**
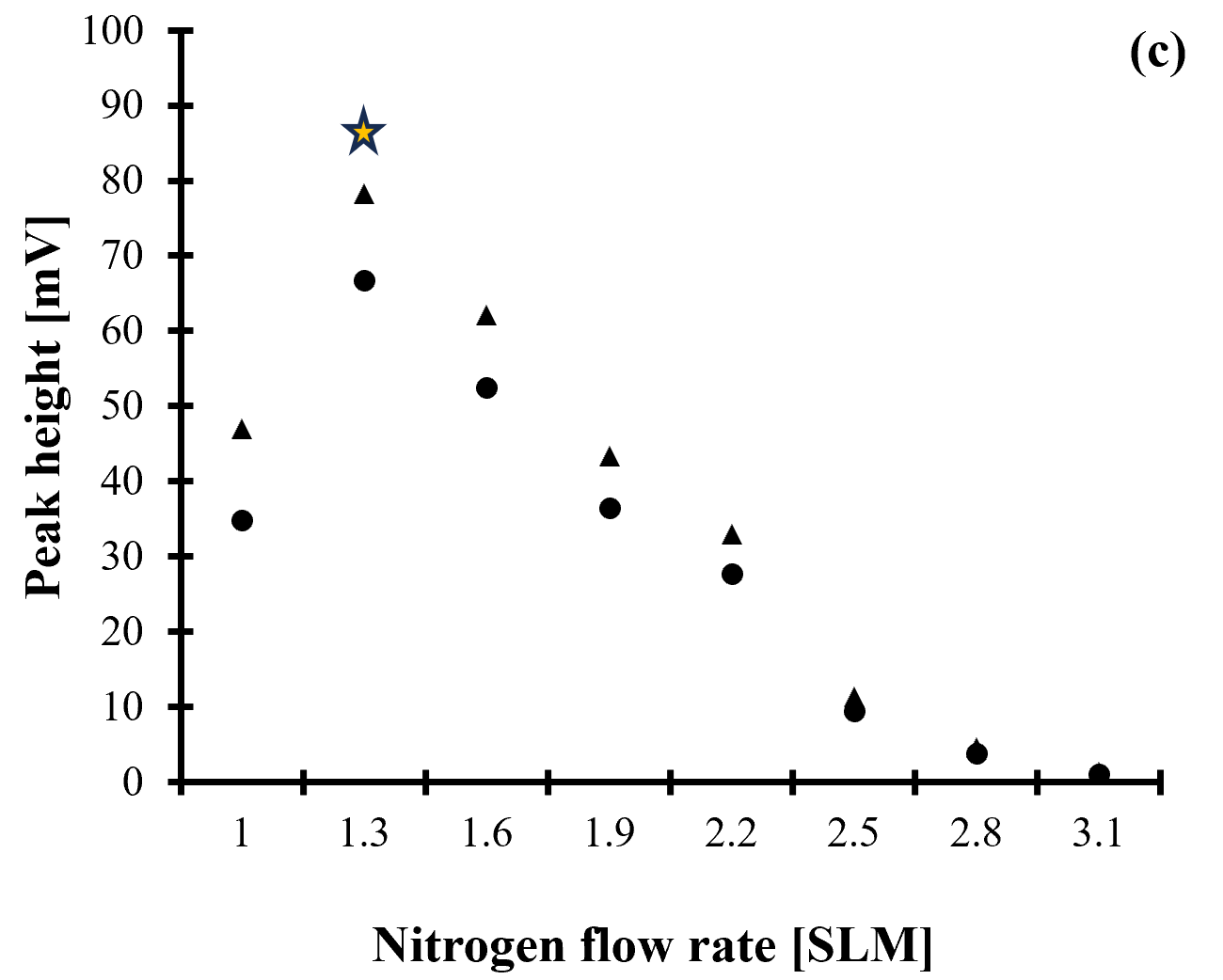

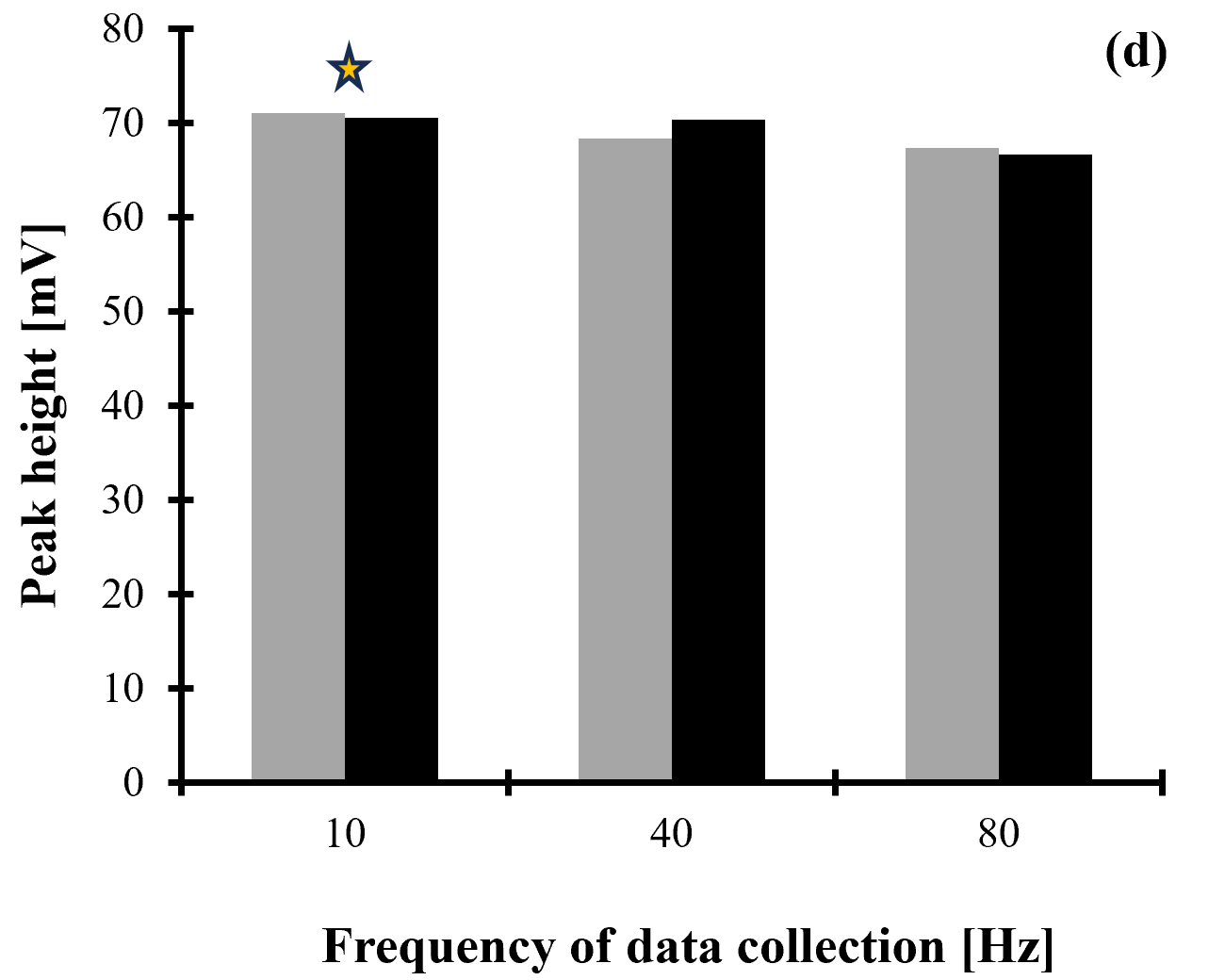
**

**
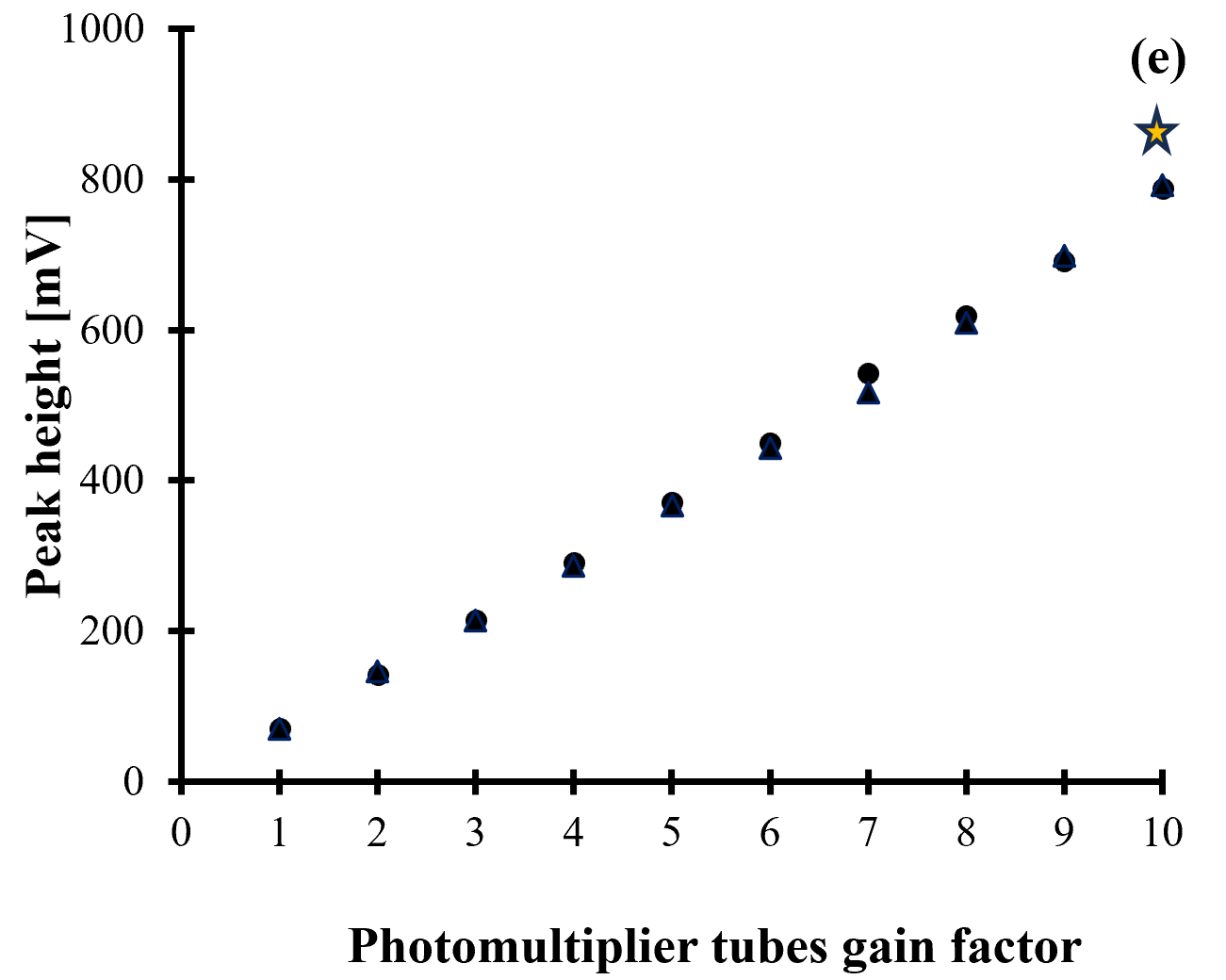
**

**Figure S2.**

**
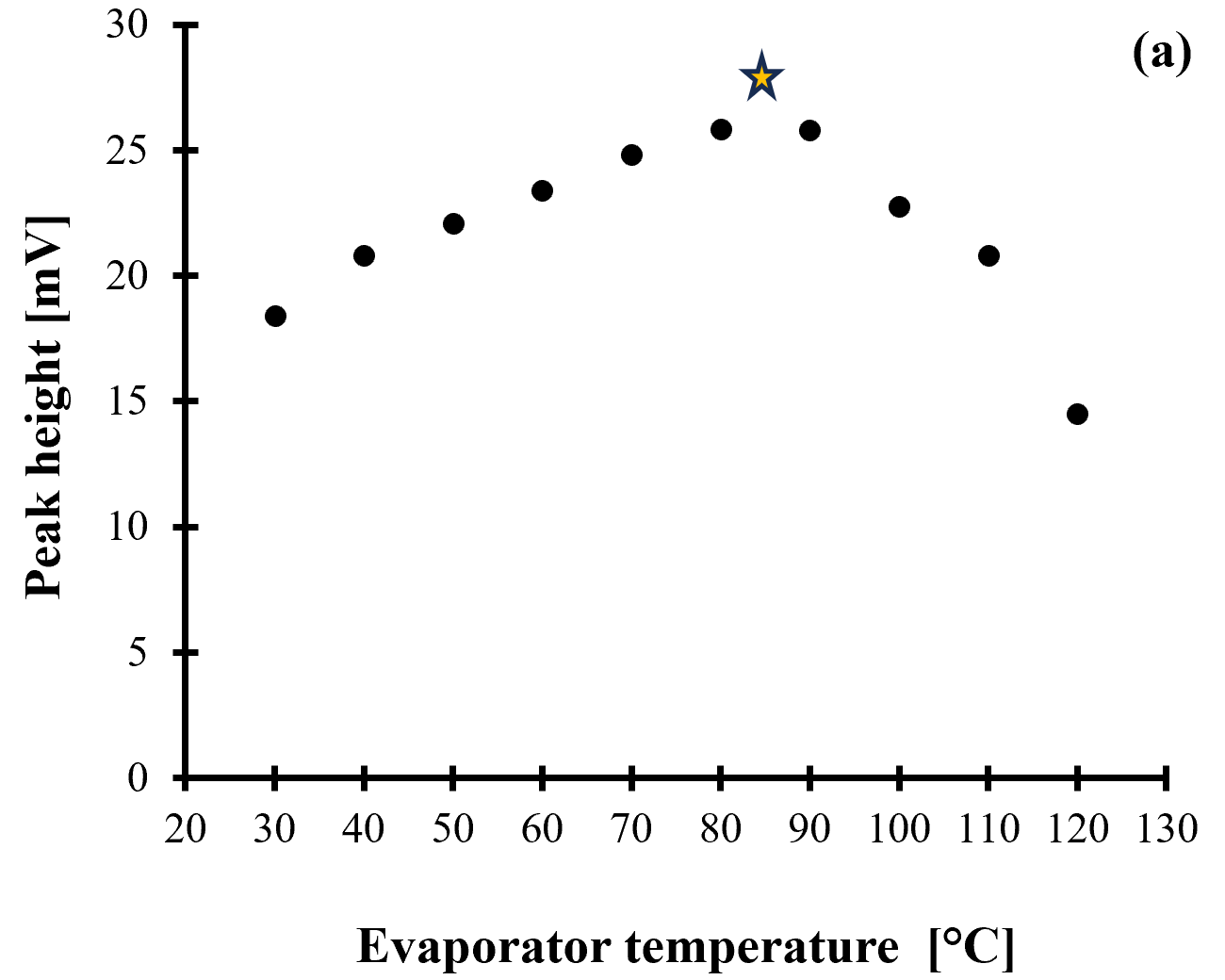

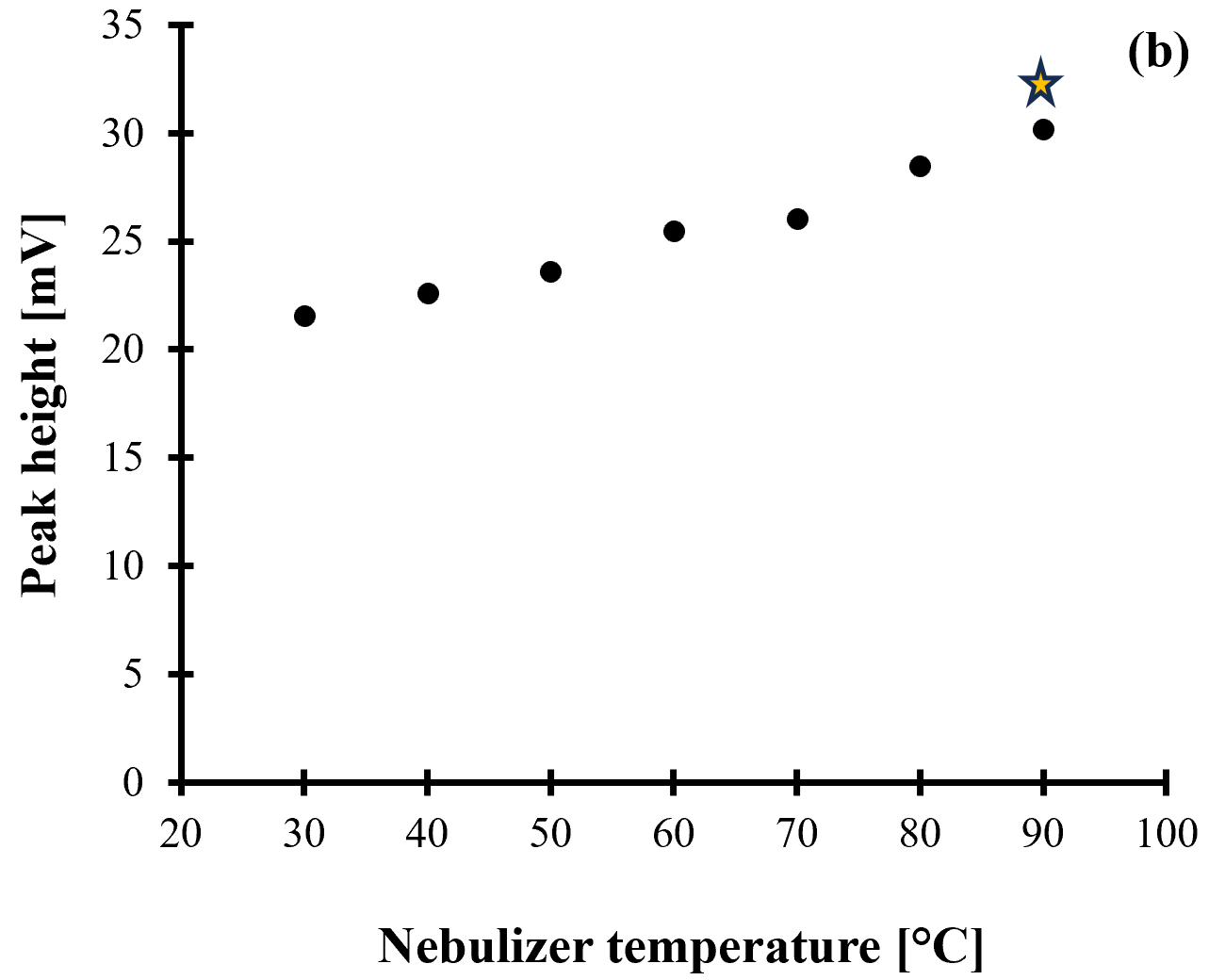
**

**
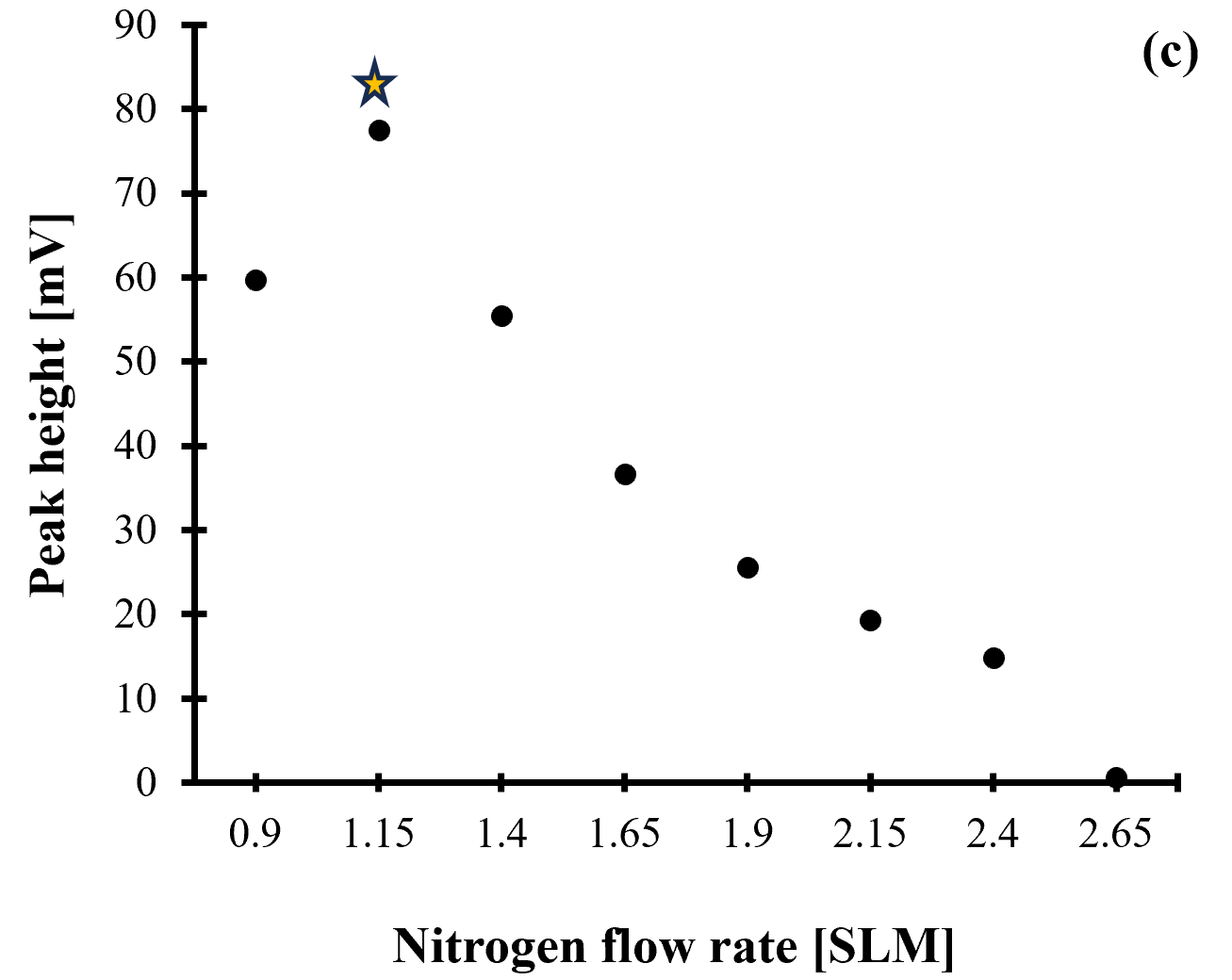

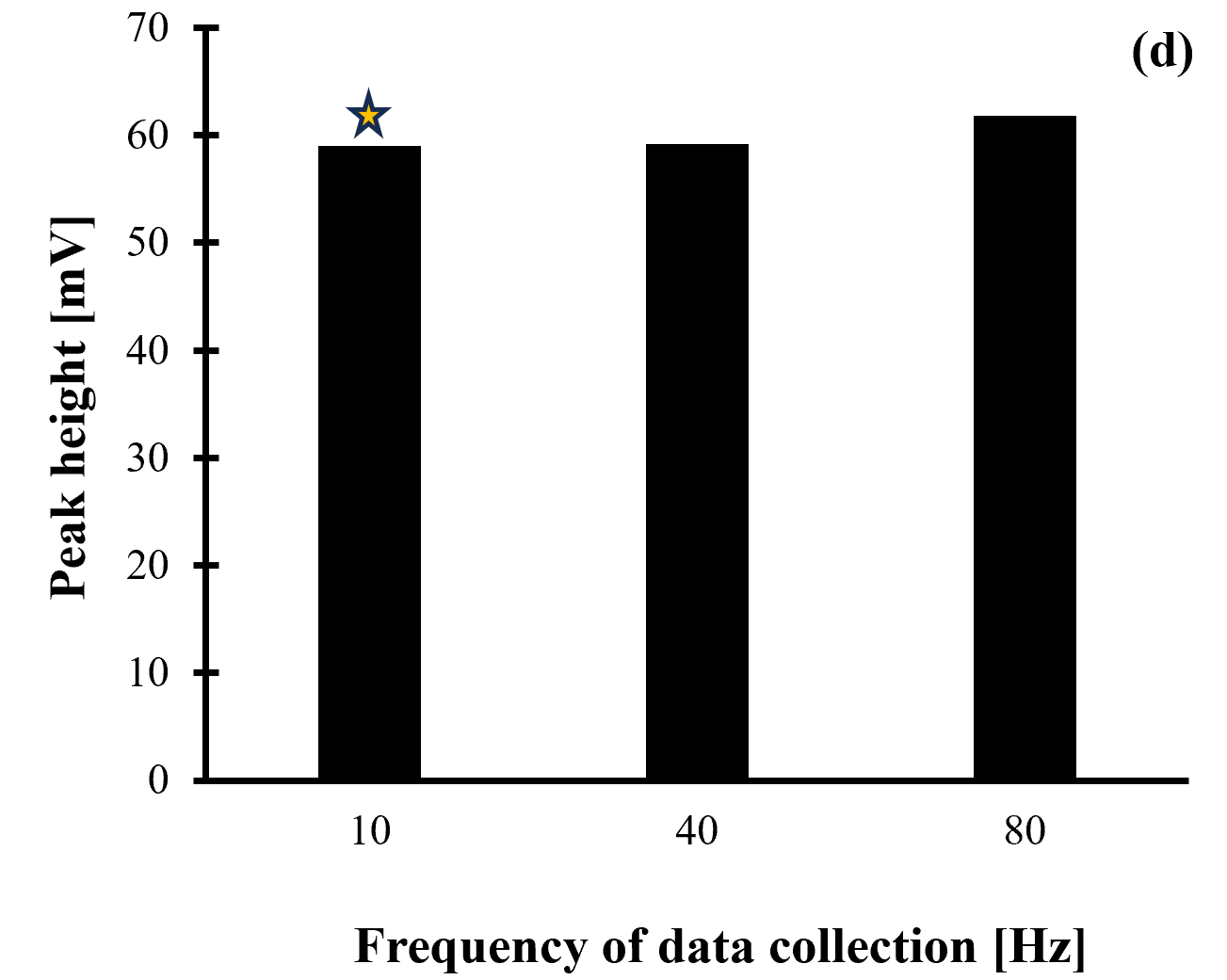
**

**
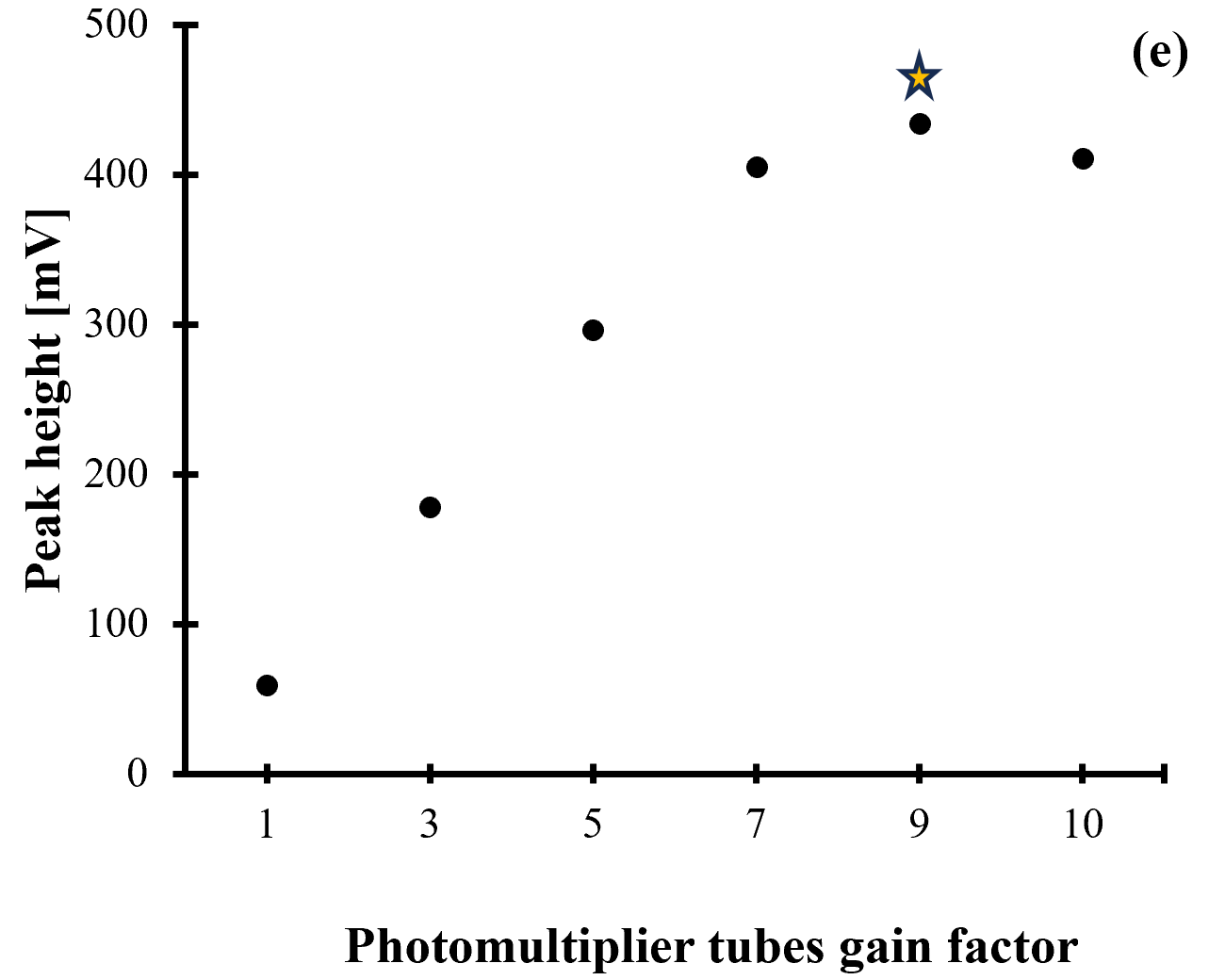

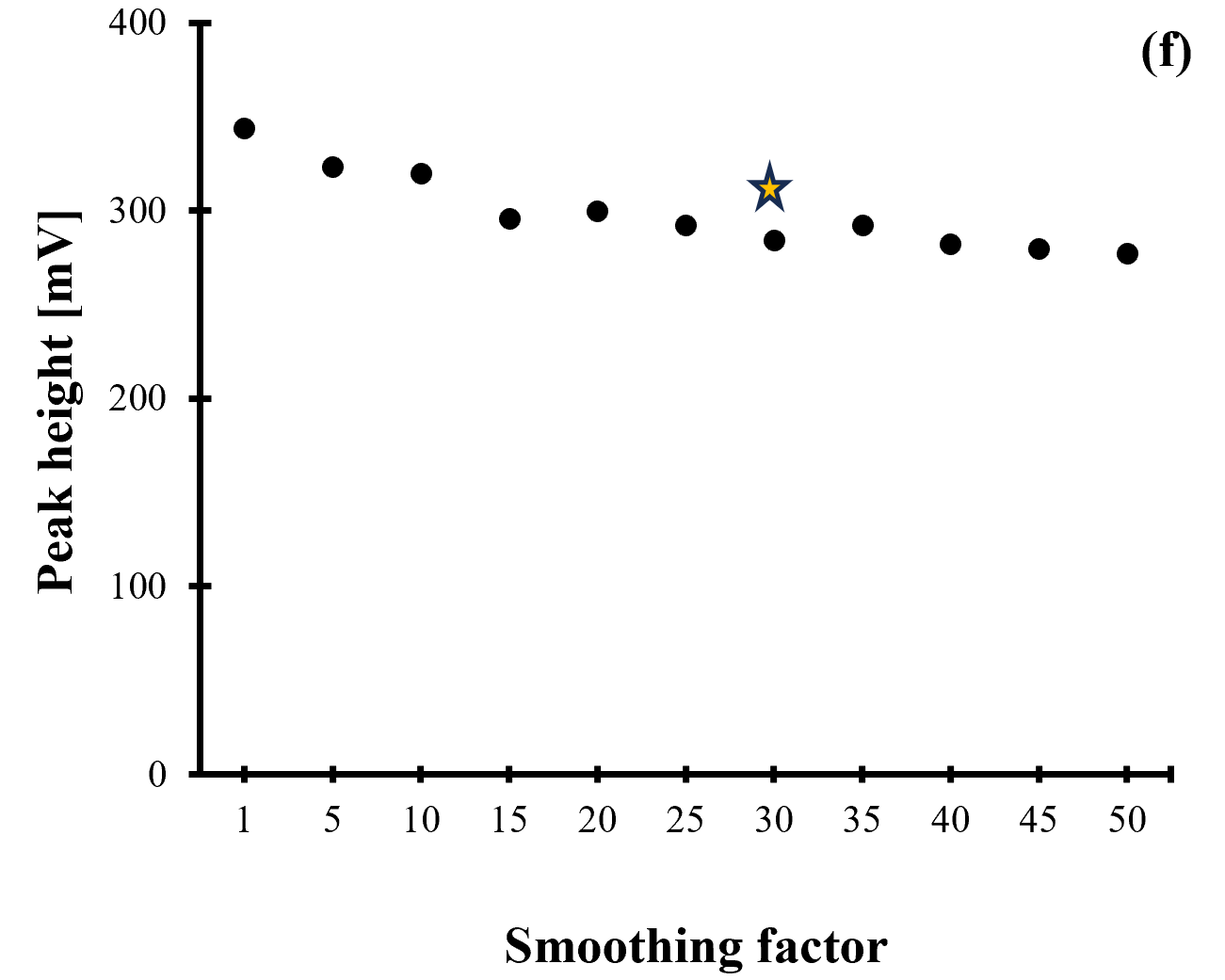
**

**Captions**

**Figure S1.** The effect of (a) evaporator temperature; (b) nebulizer temperature; (c) gas flow rate; (d) frequency of data collection; (e) signal amplification factor, expressed as the peak height of 2-HIBU (dots / grey column) and CIBU (triangles / black column). The star symbol above the measuring point indicates the optimal (nominated) value of the evaluated parameter. Chromatographic conditions were as described in section 2.6.1. The initial settings of ELSD detector were as follows: evaporator temperature 30 ˚C, nebulizer temperature 30 ˚C, gas (nitrogen) flow rate 1.60 SLM, data rate 10 Hz, PMT gain of 9, smoothing 30 (3 s).

**Figure S2.** The effect of (a) evaporator temperature; (b) nebulizer temperature; (c) gas flow rate; (d) frequency of data collection; (e) signal amplification factor; (f) smoothing factor on the response of ELSD detector, expressed as the peak height of Crn. The star symbol above the measuring point indicates the optimal (nominated) value of the evaluated parameter. Chromatographic conditions were as described in section 2.6.2. The initial settings of ELSD detector were as follows: evaporator temperature 30 ˚C, nebulizer temperature 30 ˚C, gas (nitrogen) flow rate 1.60 SLM, data rate 10 Hz, PMT gain of 9, smoothing 30 (3 s).
